# *In silico* identification of the anticataract target of βB2-crystallin from *Phaseolus vulgaris*: a new insight into cataract treatment

**DOI:** 10.3389/fchem.2024.1421534

**Published:** 2025-01-17

**Authors:** Sunday Amos Onikanni, Adewale Oluwaseun Fadaka, Tran Nhat Phong Dao, Valens Munyembaraga, Vincent Nyau, Nicole Remaliah Samantha Sibuyi, Morenike Grace Ajayi, Nguyen Thi Ai Nhung, Emmanuel Ejiofor, Basiru Olaitan Ajiboye, Minh Hoang Le, Hen-Hong Chang

**Affiliations:** ^1^ College of Medicine, Graduate Institute of Biomedical Sciences, China Medical University, Taichung, Taiwan; ^2^ Department of Chemical Sciences, Biochemistry Unit, Afe-Babalola University, Ado-Ekiti, Nigeria; ^3^ Department of Biotechnology, University of the Western Cape, Bellville, South Africa; ^4^ Graduate Institute of Integrated Medicine, College of Chinese Medicine, China Medical University, Taichung, Taiwan; ^5^ Faculty of Traditional Medicine, Can Tho University of Medicine and Pharmacy, Can Tho, Vietnam; ^6^ Institute of Translational Medicine and New Drug Development, College of Medicine, China Medical University, Taichung, Taiwan; ^7^ University Teaching Hospital of Butare, Huye, Rwanda; ^8^ Department of Food Science and Nutrition, School of Agricultural Sciences, University of Zambia, Lusaka, Zambia; ^9^ Department of Science and Innovation/Mintek Nanotechnology Innovation Centre, Biolabels Node, University of the Western Cape, Bellville, South Africa; ^10^ Department of Chemical Sciences, Bamidele Olumilua University of Education, Science and Technology, Ikere, Nigeria; ^11^ Department of Chemistry, University of Sciences, Hue University, Hue, Vietnam; ^12^ Department of Chemical Sciences, Faculty of Science, Clifford University, Owerrinta, Nigeria; ^13^ Phytomedicine and Molecular Toxicology Research Laboratory, Department of Biochemistry, Federal University Oye Ekiti, Oye Ekiti, Nigeria; ^14^ Institute of Drug Research and Development, SE Bogoro Center, Afe Babalola University, PMB5454, Ado-Ekiti, Nigeria; ^15^ Chinese Medicine Research Centre, China Medical University, Taichung, Taiwan; ^16^ Department of Chinese Medicine, China Medical University Hospital, Taichung, Taiwan

**Keywords:** βB2-crystallin, *Phaseolus vulgaris*, cataract, Schrödinger, conformation

## Abstract

**Introduction:**

Severe protein clumping in the lens can block light and lead to vision issues in cataract patients. Recent studies have linked β-crystallins, which are key proteins in the lens, to the development of cataracts. Specifically, the S175G/H181Q mutation in the βB2-crystallin gene plays a major role in cataract formation.

**Methods:**

To understand how this mutation can be activated, we utilized computational methods to predict activators from Phaseolus vulgaris. The Schrödinger platform was employed to screen bioactive compounds and simulate molecular interactions in order to analyze binding and structural changes.

**Results:**

Our results indicated that these phytochemicals are stable near S175G/H181Q.

**Discussion:**

These findings suggest novel approaches that could potentially be developed into effective anticataract medications through further refinement and additional testing, ultimately resulting in the creation of more potent agents for cataract treatment.

## 1 Introduction

Crystallins are essential proteins in the eye lens that play a vital role in directing light to the retina (Qi et al., 2016; Song et al., 2020). They constitute up to 90% of the water-soluble proteins in the human lens, supporting the stable structure of lens fiber cells throughout an individual’s life (Lou et al., 2009; Chen et al., 2018). Misfolded crystallins can lead to correctable blindness by causing clouding and blurring of the lens (Lee and Afshari, 2017). Research indicates that cataracts resulting from misfolded crystallins contribute to 10.8 million cases of blindness out of 32 million cases worldwide. There are three types of crystallins, namely, α, β, and γ, which are categorized into two families: α-crystallins and βγ-crystallins (Khairallah et al., 2015; Moreau and King, 2012). α-Crystallins, the most abundant crystallins in the lens, help maintain light refraction and act as chaperones in lens development (Fujii et al., 2012; Robinson et al., 2006). Human β-crystallins and human γ-crystallins share similar sequences, with β-crystallins further classified into acidic and basic subtypes. The basic subtypes of β-crystallins include βB-1, βB-2, and βB-3 crystallins, with βB-2 crystallins being the least altered and most soluble protein (Evans et al., 2008; Srivastava and Srivastava, 2003; Takata et al., 2018; Xu et al., 2012). A bioinformatics study of a dataset of cataract patients identified major nuclear cataract-associated mutants from βB-2 crystallins. The study revealed the impact of S175G/H181Q mutation on βB-2 crystallins, affecting the largest loop that links the β-sheet in the key Greek motif (Song et al., 2020). Furthermore, significant structural changes were observed in the βB-2 crystallin S175G/H181Q mutant, along with an increase in cataract-related modifications, such as oxidation (Song et al., 2020).

Various risk factors, such as diabetes, hypertension, smoking, and tobacco use, are linked to nuclear and cortical cataracts, which cause the degradation of crucial proteins (Mamatha et al., 2015). Recent studies have identified inhibitors such as closantel and gambogic acid that aid in preventing protein unfolding and aggregation and enhancing tetramer stability at low concentrations (Islam et al., 2022; Mishra et al., 2012). In recent years, thousands of medicinal plants have been explored for their pharmacological value, with many showing medicinal benefits (Ajiboye et al., 2022; Onikanni et al., 2022). One such plant is *Dregea volubilis*, which produces drevogenin D, a plant-derived compound used for various conditions, such as asthma, dyspepsia, and inflammation (Anjaria et al., 2002). Plant-derived compounds and herbal extracts play a crucial role in supporting the anticancer potential of medicinal plants. This evidence is backed by mechanistic approaches that highlight the antioxidant properties of these plant-based compounds. Quercetin, a flavonoid found in various fruits, vegetables, and grains, is known for its antioxidant and anticancer properties. It has been shown to prevent cataract progression and the formation of glucose-induced cataracts in the artificial aqueous humor by reducing lipid peroxidation and increasing the Na + -K + -ATPase activity (Mullins and Arjmandi, 2021; Moreno-Valdespino et al., 2020; Wang et al., 2010; Chaudhary and Sharma, 2013). Another flavone-based natural dietary polyphenol, chrysin, found in flowers and honeycombs, has been shown to prevent selenite-induced cataract formation in cultured animal lenses. Chrysin achieves this by modulating genes responsible for calcium transport, calpain formation, and apoptosis (Bellucci et al., 2014).

Additionally, legume plants such as *Phaseolus vulgaris* are rich in protein, vitamins, and phytochemicals (Papa and Gepts, 2003; Papa et al., 2005). The pharmacological significance of *Phaseolus vulgaris* in diabetes has been extensively studied, with bioactive components identified for their diabetes-regulating benefits. The consumption of *Phaseolus vulgaris* has been linked to reducing the need for cataract surgery (E et al., 2022; Pascale et al., 2018). This plant, mostly domesticated in the U.S., contains components such as stigmasterol, sitosterol, and campesterol, which contribute to its benefits (Dueñas et al., 2015; Oomah et al., 2005; Parmar et al., 2016; Ocho-Anin et al., 2010). It is also rich in bioactive chemicals and essential nutrients such as proteins, carbohydrates, dietary fiber, and fat, as well as antidiabetic polyphenol compounds and saponins (Nyau et al., 2015; Ekins et al., 2007; Onikanni et al., 2023; Umar et al., 2021; Schrödinger LLC, 2018).

Computational techniques play a crucial role in drug discovery by accelerating the process and cutting costs. Researchers have investigated various molecular targets to develop new drugs for different conditions, such as cataracts (Anjaria et al., 2002; Papa and Gepts, 2003; Papa et al., 2005). Our study aimed to understand the mechanism of action through ADMET drug-likeness analysis, molecular dynamics (MD) simulations, and principal component analysis (PCA). Specifically, we focused on identifying potential phytochemicals that could act as anticataract agents by targeting S175G/H181Q in combination with βB2-crystallin inhibition in *Phaseolus vulgaris*.

## 2 Materials and methods used for the study

### 2.1 Recovery of molecules and receptors

The library consists of 15 bioactive compounds (phytochemicals) extracted from *Phaseolus vulgaris* in a previous study by Johnson et al. (2021). The 2D structures of the ligands and reference compounds were retrieved from the database (https://pubchem.ncbi.nlm.nih.gov/). Similarly, the three-dimensional structures of the target proteins (PDB: human importin alpha 3 complex with bimax2 peptide and βB2-crystallin) were obtained from the protein library of the Scientific Collaboratory for Molecular Bioinformatics (RCSB) online (https://www.rcsb.org/).

### 2.2 Preparation of protein and grid generation

The crystal structures of the proteins of interest were generated using Schrödinger Suite version 21.3 of Maestro Wizard. This process involved minimizing the configuration using the OPLS3 force field, adding any missing hydrogen atoms, optimizing hydrogen bonds, removing water molecules, and establishing necessary disulfide bridges. Grid folders were also created to define the receptor binding sites by selecting the co-crystal ligand located within the pocket (Adekiya et al., 2022; Saeb, 2018; Lawal et al., 2023).

### 2.3 Ligand preparation

Comprehensive preparation of ligands was carried out via the latest module in Schrödinger Suite version 21.3 of Maestro, followed by OPLS3 optimization at a physiologically relevant pH range of 7.2 ± 0.2. Based on each structural molecule, potential ionization states were generated, and stereoisomers were generated by modifying the specific chirality while keeping the other stereoisomers constant (Lawal et al., 2023).

### 2.4 Toxicological prediction and ADME properties of the identified molecules

QikProp Schrödinger Suite version 21.3 of Maestro tools was used to analyze the ADME properties and toxicological potency of the lead molecules (Anand et al., 2019).

### 2.5 Free binding energy determination

Schrödinger Suite version 21.3 of Maestro was utilized to explore the use of Prime MM-GBSA software in calculating the binding energy potential of receptor–ligand complexes. This was done to determine the stability of these complexes. The OPLS3 force field and VSGB solvent model were chosen as the default force field and solvent model for free energy binding calculations, respectively (Johnson et al., 2021). The OPLS3 force field and VSGB continuum solvent model were selected, with default settings for other options (Tewari et al., 2019). The formula used to determine the binding energy (ΔGbind) of each ligand with LASV nucleic acids is as follows: ΔE + ΔGsolv + ΔGSA = ΔGbind. The expression ΔE = E_complex_ − E_protein_ − E_ligand_ represents the reduced energies of the protein–inhibitor complex, protein, and inhibitor, respectively. Furthermore, Gsolv (_complex_) = ΔGsolv – Gsolv (_complex_) – Gsolv (_protein_) – Gsolv (_ligand_), where Gsolv (_complex_), Gsolv (_protein_), and Gsolv (_ligand_), represent the solvation free energies of the complex, protein, and inhibitor, respectively. ΔGSA = GSA (_complex_) – GSA (_protein_) – GSA (_ligand_), which represents the surface area energies of the complex, protein, and inhibitor, respectively (Tewari et al., 2019).

### 2.6 Trajectory point analysis and molecular dynamics simulations

The molecular mechanism of the target protein was investigated on the Windows-x64 platform using Schrödinger Suite 21.3, Maestro version 12.5.137, and MM share version 5.7.137. Techniques for trajectory analysis and MD preparation had been previously developed (Jeevanandam et al., 2021). The docked complexes underwent molecular modeling with an OPLS 2005 force field and the Desmond module of Schrödinger software. The receptor–ligand complex was placed in an orthorhombic box using a transferable intermolecular potential and a 3-point water model. Salt and chloride ions were added to mimic physiological conditions and neutralize the overall charge. Temperature and pressure were maintained at 310°C and 1.01325 bar, respectively, using an American-made Martyna–Tobias–Klein barostat and Nose–Hoover thermostat. Simulated relaxation was carried out in an NPT ensemble, considering the number of atoms, pressure, and timescale. Long-range electrostatic interactions were calculated using the particle mesh Ewald approach during the MD simulation. A 100-ns MD simulation study was conducted with a 1000-frame trajectory sampling interval. MS-MD trajectory analysis and a simulation interaction diagram were used to assess and present the simulation results. To ensure consistency, the MD analysis was performed in duplicate. Data were plotted using Origin Pro version 9.

## 3 Results

**FIGURE 1:**
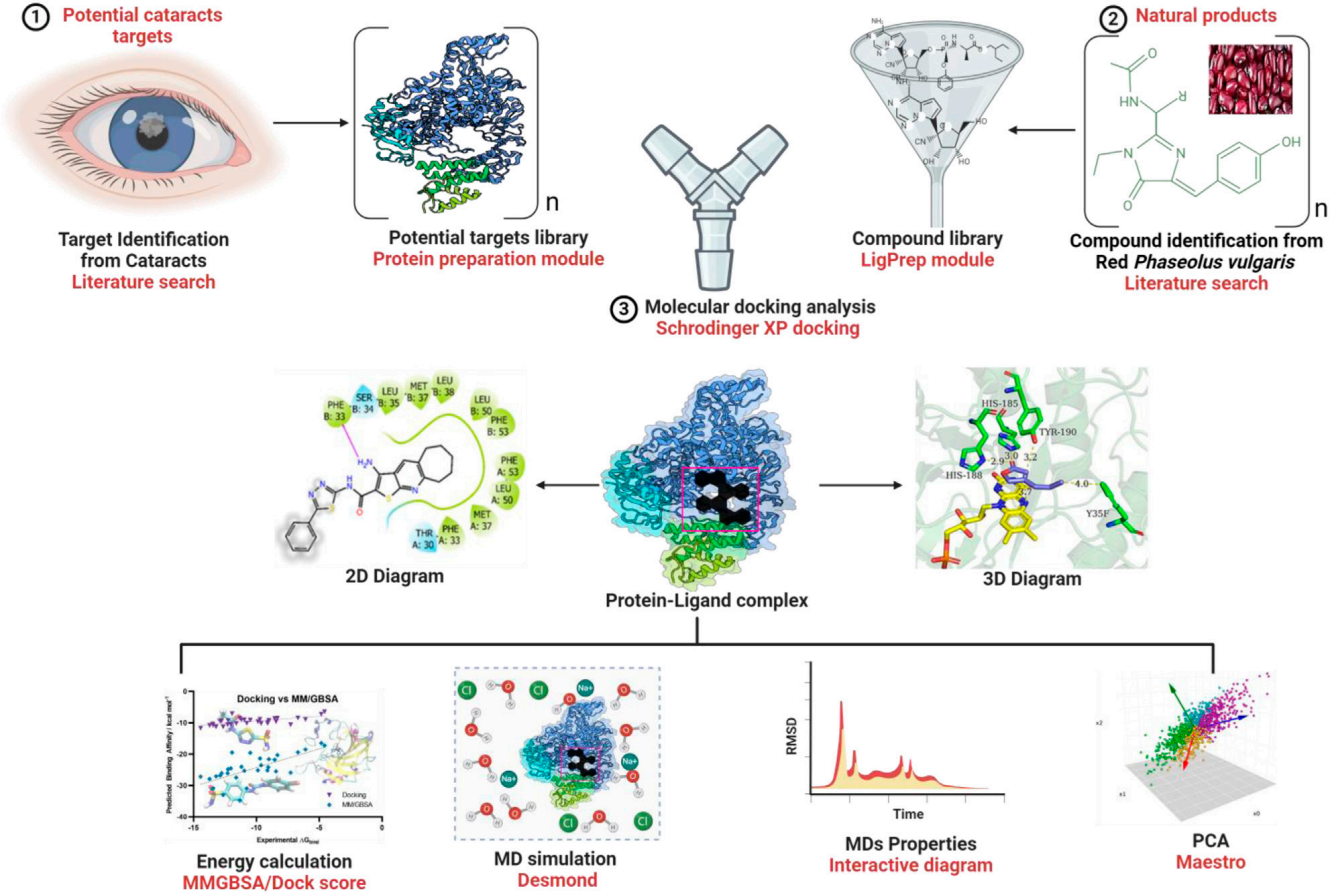
The workflow representation from the research study.

**FIGURE 2:**
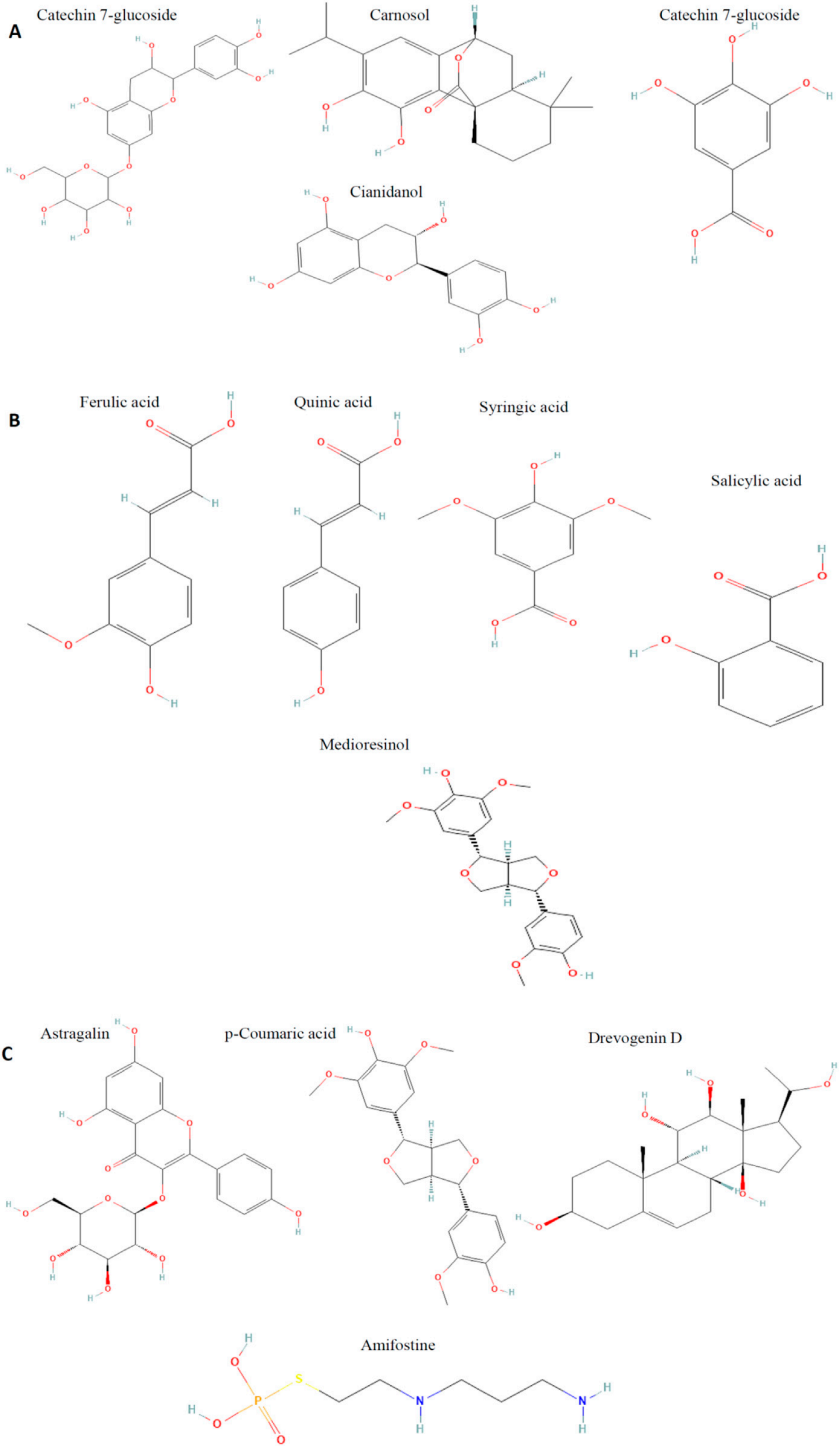
**(A-C)** Structural composition of the molecules previously identified and characterized in our study of *Phaseolus vulgaris*, along with reference molecules.

The graphical chart of the *in silico* study is presented in Figure 1 to provide an overview of the research study. Physicochemical properties such as alogP, atomic number, ring number, H-bond acceptors, and H-bond donors are crucial functions in the applicability domain. We analyzed the distribution of these properties in all the training sets of the predictive models, and the structural compositions of the compounds of interest are depicted in Figures 2A–C. Furthermore, the importance of pharmacokinetics in predicting the absorptivity, distribution, metabolism, and excretion of any drug is essential for its safety and therapeutic effectiveness. Therefore, the therapeutic agent requires the presence of H-bond acceptors and H-bond donors for membrane transport, and the interactions of drug proteins, rotatable bonds, and aqueous solubility are consistent among the compounds of interest compared with the reference molecules, as shown in Table 1. Moreover, one of the most critical properties in drug discovery is the compound’s solubility as it significantly impacts various drug properties, such as biological activity, toxicity, pharmacokinetic potential, and *in vivo* potency. These parameters are determined during different stages of drug discovery and development. Therefore, the preclinical stage of any drug discovery requires solubility, especially during the structural optimization process. Our study revealed that the molecules of interest are more soluble than the reference ligands, as shown in Table 1.

**TABLE 1:** Bioabsorbability and pharmacokinetic analyses of the molecules of interest were performed with SWISSADME, while the medicinal chemical effects were evaluated using admetSAR online servers.

		Water solubility		Pharmacokinetics		Drug-likeness	
ID	Log S (ESOL)	Log S (Ali)	GI absorption	BBB permeant	P-gp substrate	Lipinski	Bioavailability
QUI	Highly soluble	Highly soluble	Low	No	No	0 violation	0.56
GAL	Very soluble	Soluble	High	No	No	0 violation	0.56
CATG	Very soluble	Soluble	Low	No	No	2 violations	0.17
SYR	Very soluble	Soluble	High	No	No	0 violation	0.56
CAT	Soluble	Soluble	High	No	No	0 violation	0.55
FER	Soluble	Soluble	High	Yes	No	0 violation	0.85
MED	Soluble	Soluble	High	No	Yes	0 violation	0.55
PCO	Soluble	Soluble	High	Yes	No	0 violation	0.85
SAL	Soluble	Soluble	High	Yes	No	0 violation	0.85
KAE	Soluble	Moderately soluble	Low	No	No	2 violations	0.17
CAR	Moderately soluble	Moderately soluble	High	Yes	Yes	0 violation	0.55
DRE	Soluble	Soluble	High	No	Yes	0 violation	0.55
AMI	Highly soluble	Highly soluble	High	No	No	0 violation	0.55

Keywords**:** QUI, quinic acid; GAL, gallic acid; CATG, catechin glucoside; SYR, syringic acid; CIA, cianidanol; FER, ferulic acid; MED, medioresinol; PCO, p-coumaric acid; SAL, salicylic acid; AST, astragalin; CAR, carnosol; DRE, drevogenin D; AMI, amifostine.

Furthermore, our findings revealed the hit molecules compared with reference compounds (Drevogenin D and Amifostine), an inhibitor of the Pgp substrate, the Pgp substrate had no inhibitory effect on any of the molecules of interest. Surprisingly, few of the bioactive compounds extracted from *Phaseolus vulgaris* showed little intestinal absorption, in contrast to the reference agonists, suggesting that most of these compounds demonstrated significant potency as ATP-dependent drug efflux pumps for ADME drugs through substrate specificity.

**FIGURE 3:**
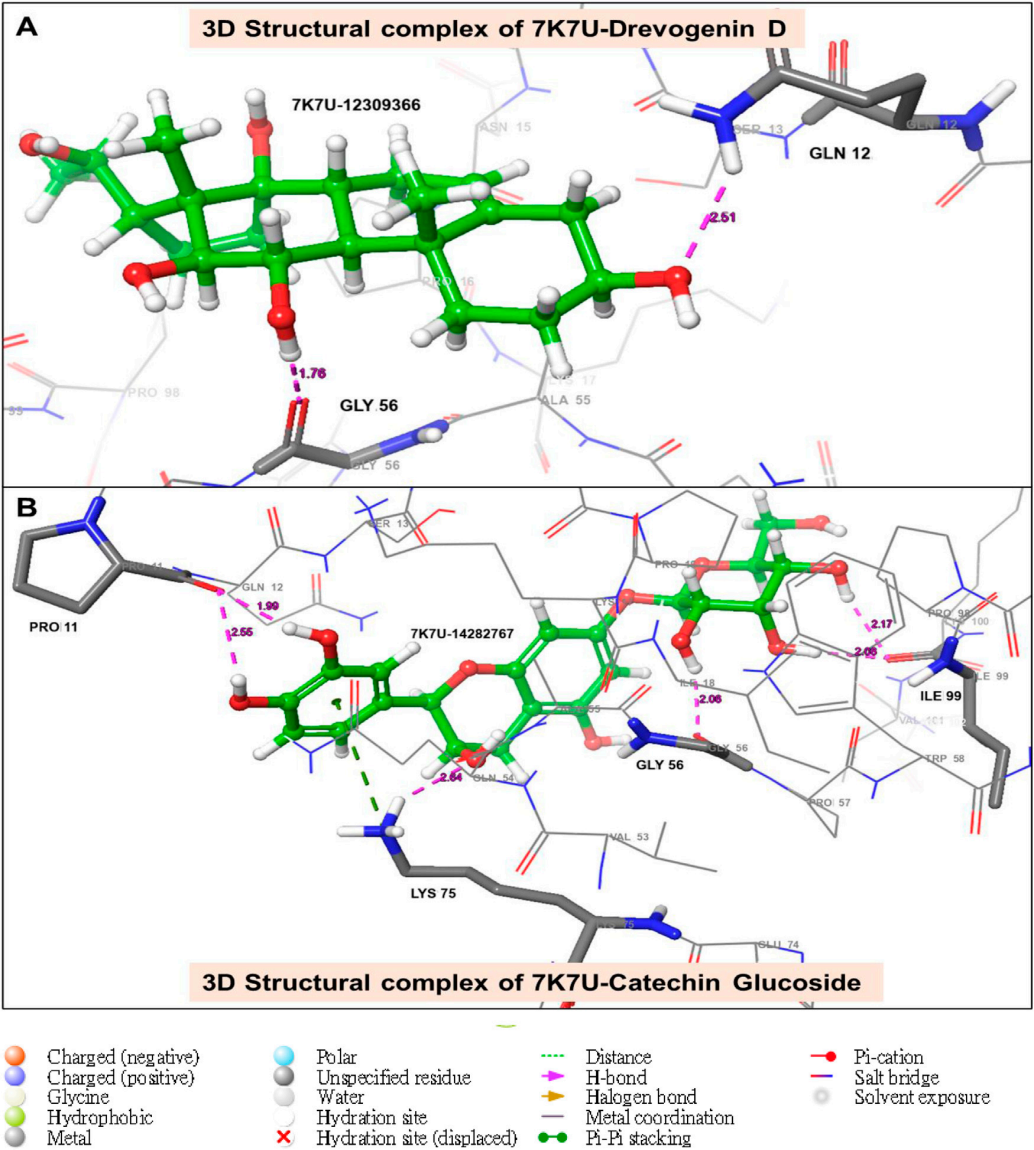
The green stick model illustrates the interaction between the molecular complexes of the crystal structure of BetaB2-crystallin and Drevogenin D, as well as the crystal structure of BetaB2-crystallin and Catechin glucoside at the active site. The model uses colors to represent negative, positive, and neutral charges for the corresponding binding site residues.

**FIGURE 4:**
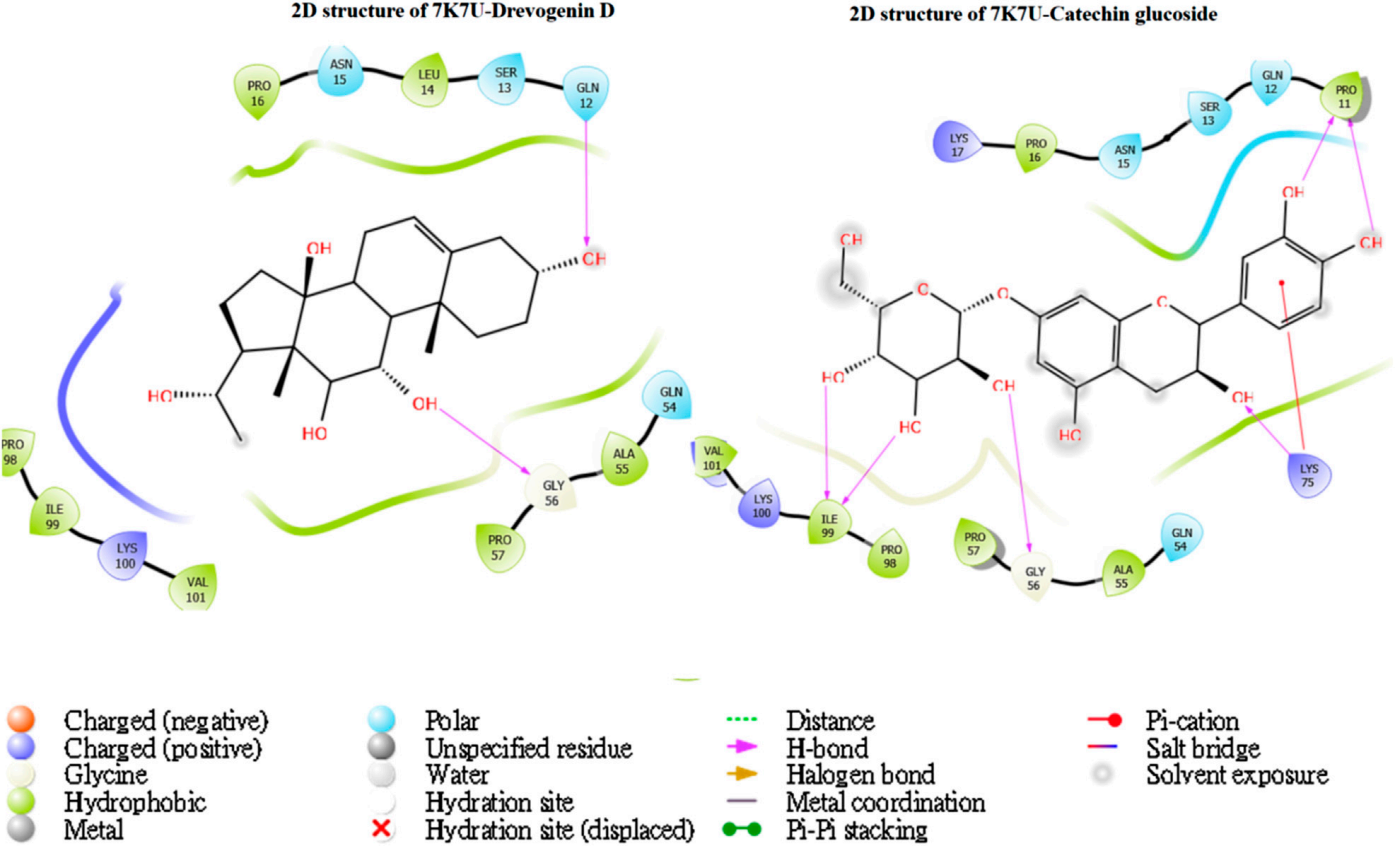
The green stick model illustrates the interaction between the molecular complexes of human importin alpha 3 activator and catechin glucoside at the active site. 2D visualization uses red, blue, and white colors to represent the corresponding binding site residues.

**FIGURE 5:**
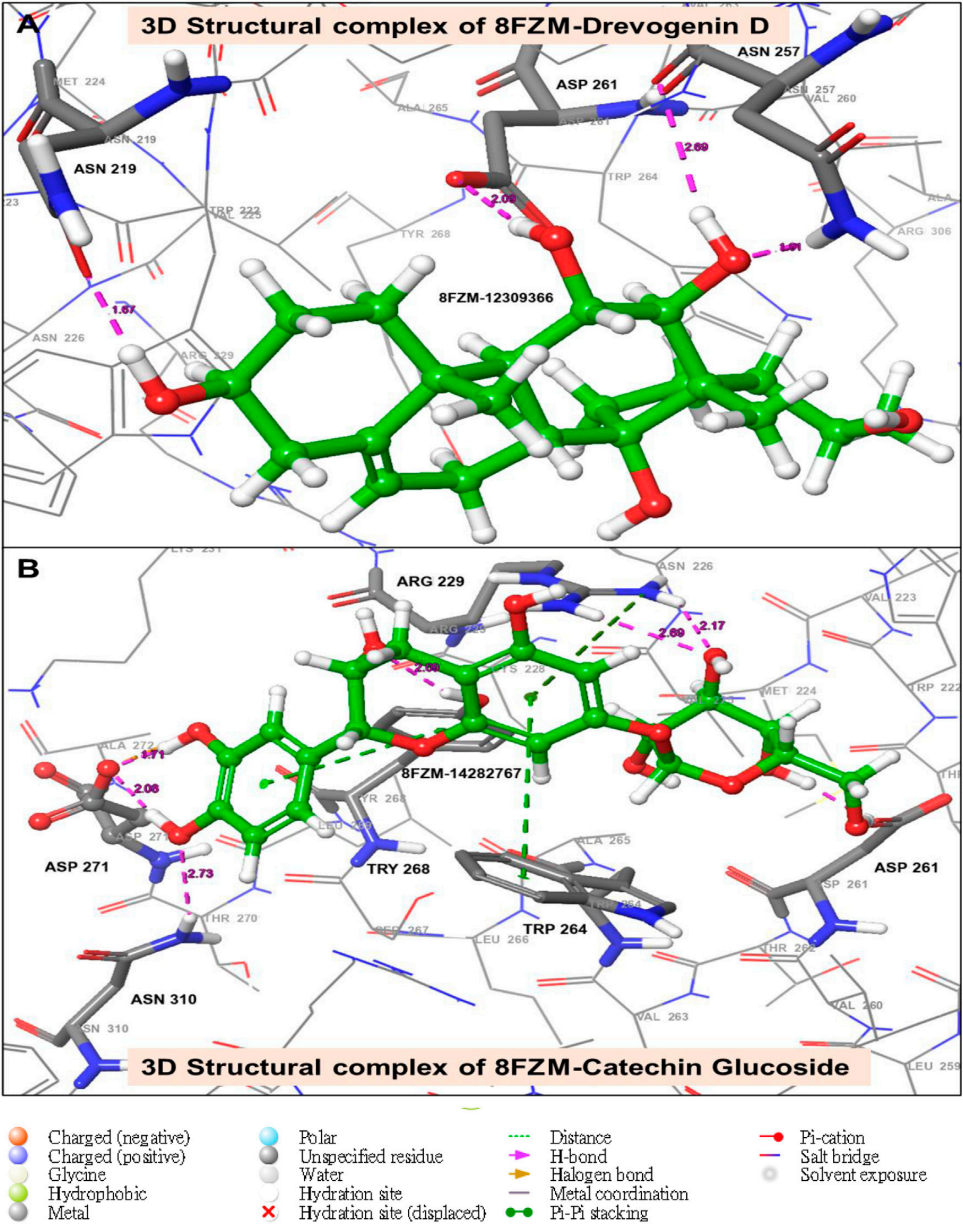
The green stick model illustrates the interaction between the molecular complexes of human importin alpha 3-activator and Drevogenin D, as well as the crystal structure of human importin alpha 3-activator and Catechin glucoside at the active site. The 3D representation shows negative, positive, and neutral charges for the binding site residues.

**FIGURE 6:**
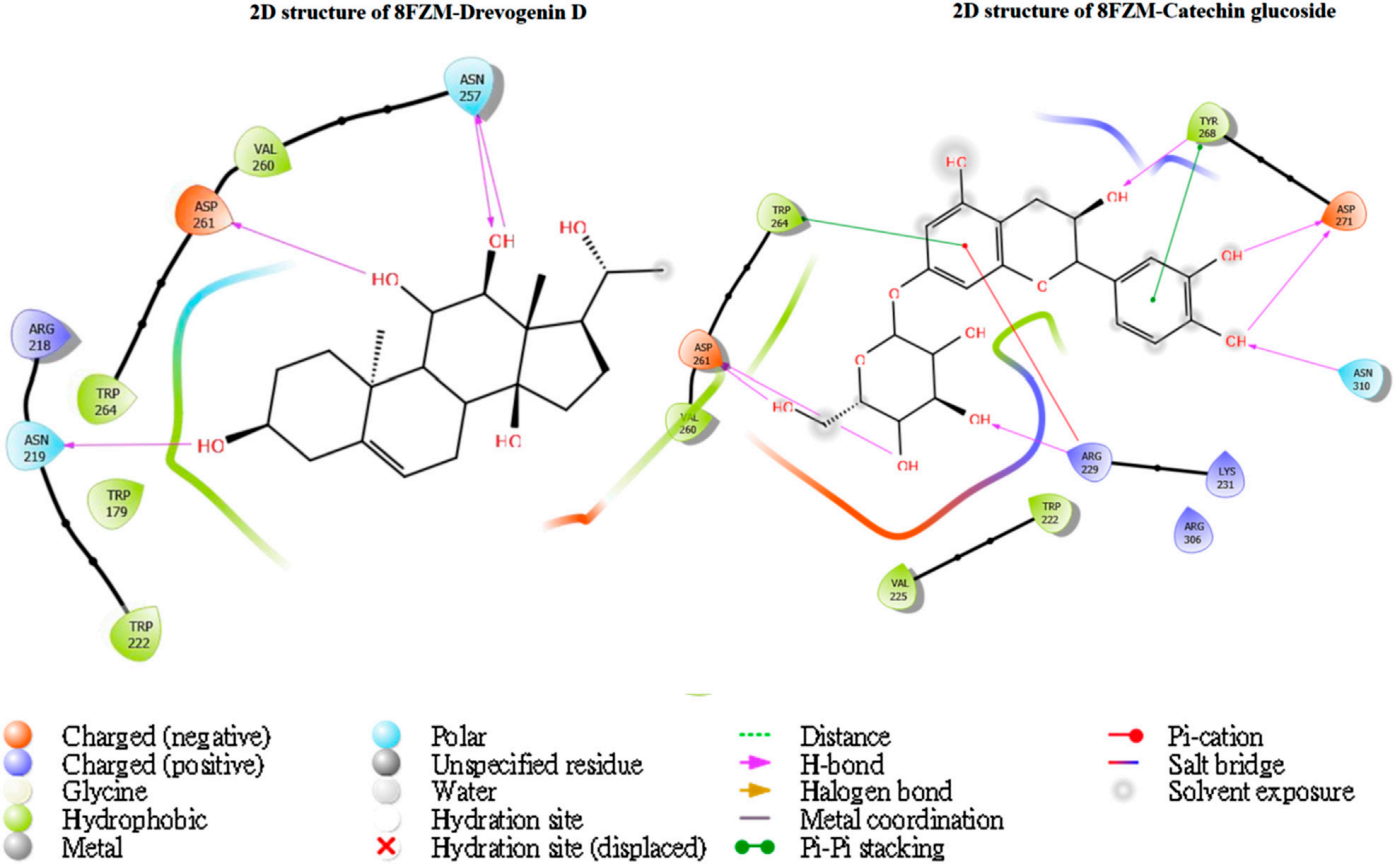
The green stick model shows the interaction between the βB2-crystallin activator and catechin glucoside molecular complexes at the active site. 2D visualization uses red, blue, and white colors to represent the corresponding binding site residues.

The utilization of lead-extracted bioactive compounds from *Phaseolus vulgaris* yielded similar results, with the crystal structure domains of the two receptors of interest (human importin alpha 3 and βB2-crystallin) producing comparable docking scores of 7.14 kcal/mol and −7.67 kcal/mol, respectively, compared to the standard docking scores of −4.54 kcal/mol and 3.97 kcal/mol, respectively. The related molecules were identified as targets of interest through docking analysis, as presented in Table 2. Additionally, one of the lead compounds demonstrated favorable interactions with ASP271, ARG229, and TYR268 in the N-terminal domain receptor of human importin alpha 3. This compound engaged in hydrogen bonding with the atoms, exhibited a π–π cation interaction with ARG229, and formed a salt bridge between TRP264 and TYR268 within the domain receptor of the human importin alpha 3 activator complex. Conversely, ILE199, PRO11, LYS75, and GLY56 participated in hydrogen bonding in the N-terminal domain receptor βB2-crystallin with the same compound, with LYS75 forming π–π cation interactions, as detailed in Table 2. Overall, the interactions indicated that the ligands influenced the flexibility of the target, while the receptor residues of the target displayed strong, detectable binding affinities. The intermolecular potential output shows quantitative affinities for binding substances that support the target’s flexibility and receptor residues. Consequently, the target proteins were analyzed through 3D and 2D molecular docking to identify the compound that occupies the enzyme’s active site. This is illustrated in Figures 3–6.

**TABLE 2:** Docking results of the complexes with the receptors.

TR	LGs	D-Score	MMGBSA	# H-bond	Pi-cat	Others
8FZM	Catechin glucoside	-7.14	-50.29	2(ASP261), 2(ASP271) ARG229, TYR268, and ASN310	ARG229	TRP264 and TYR268
	Garlic acid	-4.70	-8.86	2(HIS134), GLU122, and LYS120	0	0
	Astragalin	-4.50	-32.77	2(ILE124), 2(HIS134), and GLU122	0	0
	Medioresinol	-3.13	-37.81	GLU122 and ILE124	0	0
	Drevogenin D	-4.54	-30.01	2(ASN257), ASN219, and ASP261	0	0
	Amifostine	-4.04	-16.51	2(ASP126), 2(ASP125) GLU122, and ILE124	0	ASP126
7K7U	Catechin glucoside	-7.68	-54.98	2(ILE199), 2(PRO11) LYS75, and GLY56	LYS75	0
	Garlic acid	-6.22	-22.18	GLU105	0	0
	Astragalin	-5.62	-0.32.69	ILE99 and LYS75	0	0
	Medioresinol	-4.24	-35.19	GLN146	0	0
	Drevogenin D	-3.97	-32.65	GLY36 and GLN12	0	0
	Amifostine	-3.42	-12.61	GLN146, GLU105, SER103, and SER147	0	0

Note: 8FZM, crystal structure of human importin alpha 3 in complex with the Bimax2 peptide; 7K7U, crystal structure of βB2-crystallin.

## 4 Discussion

Understanding the interaction between receptors and their ligands is crucial for the pharmaceutical and functional food industries, as well as for scientific research aiming to uncover the root causes of many diseases worldwide. The emergence of bioinformatics has provided a platform for investigating disease mechanisms at the molecular level using computational methods (Saeb, 2018; Gogoi et al., 2016). The interaction between a protein and its ligand is essential for developing structure-based drugs, and there is a growing interest in small molecules as alternative treatments for cataracts. However, the current clinical pipeline may not be adequate to prevent disease recurrence, transmission, or resistance to existing treatments (Rampogu et al., 2018; Lamichhane et al., 2023; Lim et al., 2018; Gopal et al., 2016). Therefore, the search for new and effective anticataract medication is imperative. This study utilized a computational approach to evaluate the therapeutic potential of phytochemicals from *Phaseolus vulgaris* in reducing cataracts. Interestingly, the ADMET potential estimation of the identified phytochemicals from *Phaseolus vulgaris* revealed their characteristics compared to reference molecules in terms of heavy atoms, aromatic heavy atoms, rotatable bonds, H-bond acceptors, and H-bond donors, as shown in Table 1.

The docking score can indicate the ability of a protein–ligand complex for activation or suppression Zheng et al., 2022; Agu et al., 2023. Table 2 presents significant results with no breaches of Lipinski’s rule of five in the categories of compound-screened phytochemicals from *Phaseolus vulgaris*. This suggests that the identified ligands show therapeutic potential due to their binding pockets and rift domains in the state 1 receptor complex of human importin alpha 3 and βB2-crystallin with most compounds. By comparing the intermolecular interaction profile of the higher binding molecules to that of reference molecules, a deeper understanding of the mechanistic relationships was obtained. Catechin glucoside, with a higher docking score, emerged as one of the top contenders. Furthermore, gastrointestinal absorption of a drug is crucial for its possible delivery to the target site [Hua, 2020; Han et al., 2024; Stillhart et al., 2020]. Therefore, during gastrointestinal absorption, most of the ligands of interest from *Phaseolus vulgaris* compound-screened phytochemicals compete with reference molecules. As shown in Table 2, the gastrointestinal absorption of the ligands was comparable to that of the reference molecules.

Additionally, Table 1 shows that the target ligands inhibited the Pgp substrate in a manner similar to that of the reference compounds, which were hypothesized to be Pgp substrate inhibitors. This helps clarify why the chemical found in Table 1 can act as an ATP-dependent drug efflux pump for ADME drugs with strong potency through substrate specificity. In a similar manner, the critical ligands were docked with receptors to elucidate the therapeutic efficacy of phytochemicals screened from *Phaseolus vulgaris*. Table 2 demonstrates the validation of interactions with the majority of critical amino acid residues, suggesting that these amino acids engage with the ligands at the binding site, which may indicate that the selected hit molecule is potentially safe for use as an anticataract drug. In comparison with the reference molecules, which exhibited docking scores of −4.54 kcal/mol and 3.97 kcal/mol, the crystal structure domains of the two receptors of interest (human importin alpha 3 and betaB2-crystallin) yielded docking scores of −7.14 kcal/mol and −7.67 kcal/mol respectively, suggesting a comparable influence on the efficacy of compound-screened phytochemicals from *Phaseolus vulgaris*.

Moreover, one of the lead compounds interacts well with the human importin alpha 3 N-terminal domain receptors ASP271, ARG229, and TYR268. The H-bond with the atoms revealed a π–π cation interaction with ARG229 and a salt bridge between TRP264 and TYR268 with the human importin alpha 3 activator complex domain receptor. Conversely, the N-terminal domain receptor βB2-crystallin involved ILE199, PRO11, LYS75, and GLY56 in hydrogen bonding with the same molecule, indicating that LYS75 produced a π–π cation connection, as shown in Table 2.

**FIGURE 7:**
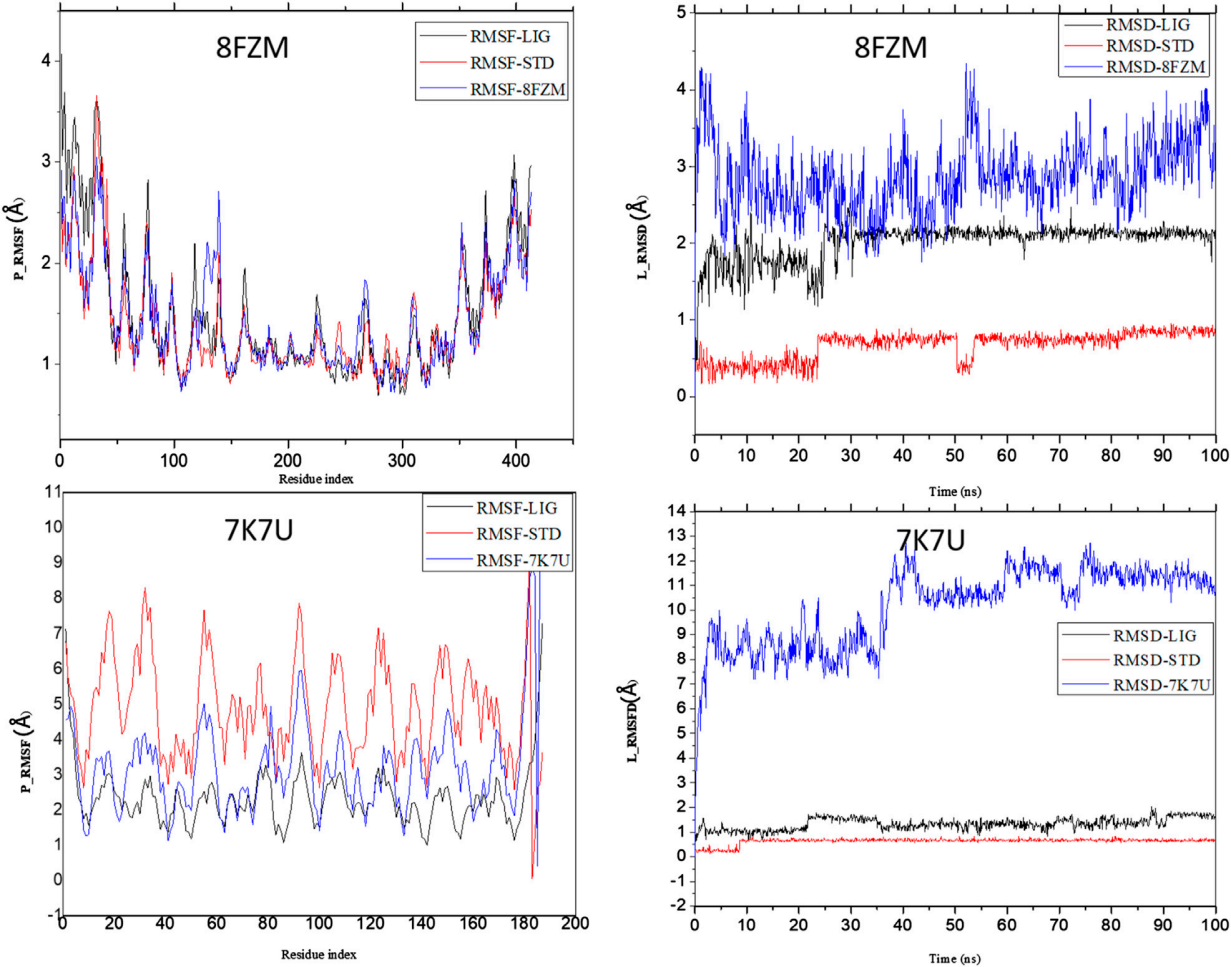
Analysis of the molecular dynamics (MD) simulations of both the human importin alpha 3 activator complex and βB2-crystallin activator. P-RMSF graphical illustration and RMSD diagram. The structural dynamics (MD) data were analyzed using Schrödinger version 2021_1 software.

**FIGURE 8:**
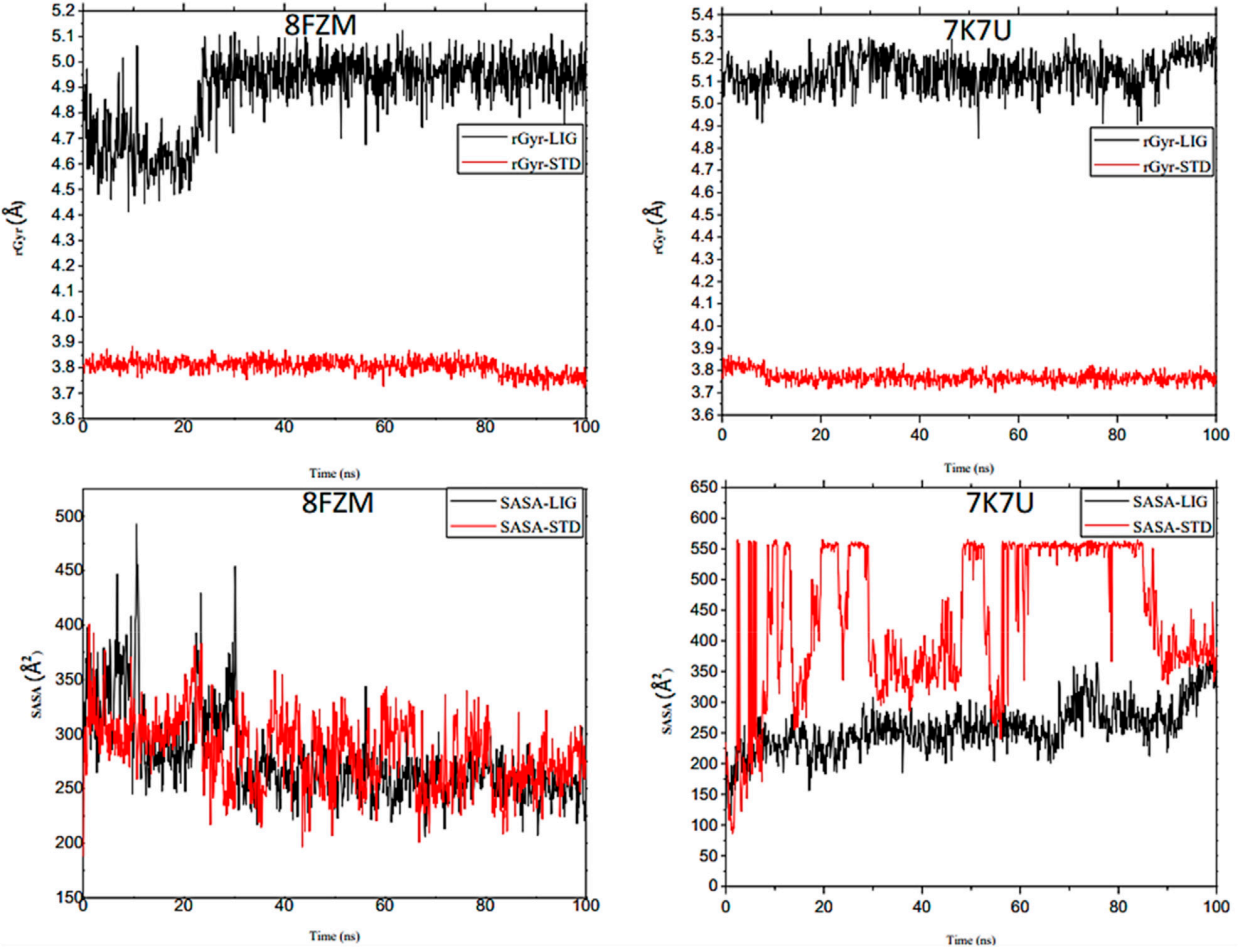
Analysis of the molecular dynamics (MD) simulations of both the human importin alpha 3 activator complex and βB2-crystallin activator. rGyr (radius of gyration) graphical illustration and SASA diagram. The structural dynamics (MD) data were analyzed using Schrödinger version 2021_1 software.

The intermolecular potential exhibits quantifiable affinities for both receptor residues and drugs that augment the adaptive ability of the target. The target proteins were subjected to three-dimensional and two-dimensional molecular docking experiments with the relevant compounds to discover the compounds of interest positioned in the enzyme’s active region, as depicted in Figures 3–6. Analyzing the atomistic level changes in receptor binding pockets over time is another critical aspect of molecular simulation analysis. The MD simulations were performed on important receptors and complexes with molecules to investigate the effect of phytochemicals screened from *Phaseolus vulgaris* on their effectiveness as activators of the mutant gene. The active site of the protein was balanced using Schrödinger Suite Maestro v21.3. Several molecular dynamics characteristics were investigated, including root mean square deviation (RMSD), root mean square fluctuation (RMSF), radius of gyration (rGyr), molecular surface area (MolSA), pressure swing adsorption (PSA), and solvent-accessible surface area (SASA). Table 3 shows the mean ± SEM of the protein binding complex and the interpreted values of the deviation plots. Throughout the 100-ns simulation period, the deviation patterns of the two receptor complexes (human importin alpha 3 activator complex and with βB2-crystallin activator) show similar movement, with catechin glucoside emerging as the dominant component, as shown in Table 3. Similarly, Figure 7 reveals that the root mean square deviation alterations were observed in all the patterns of the human importin alpha 3 activator complex at 10–85 ns. Additionally, the unraveling processes in the structure are revealed by the receptor’s compactness, perturbations, and folding status in gyration (rGyr) values that indicate changes in compactness. The average plot in Figure 8, which converges and demonstrates strong system stability, illustrates a consistently stable system over a simulation time of 23–100 ns. Table 3 presents the deviation plots and means ± SEMs for the receptor binding complex. The SASA values remain stable despite a conformational change in the target protein. However, the major suggestion from this research revealed that the increased surface area was probably exposed to the solvent due to the unfolding of the receptor, which suggests whether the SASA was folded or unfolded at the specific site of interest. Additionally, the change in the solvent-accessibility surface area from 5 ns to the end of the simulation period was demonstrated by the crystal structure of human importin alpha 3. This structure was used to analyze the protein surface’s interactions with the surrounding solvent molecules, as shown in Figure 8.

**TABLE 3:** Molecular dynamics simulation properties of native proteins and molecules of interest.

Receptor	Name	RSMF	RSM D	rGyr	SASA	MolSA	PSA
8FZM	Catechin glucoside	1.496±0.67	2.013±0.25	4.884±0.14	276.40±39.10	377.11±2.92	363.75±711
	Drevogenin D	1.397±0.51	0.761±0.18	3.805±0.02	283.57±1.92	315.57±1.92	171.18±6.64
	8FZM-alone	1.441±0.51			2.861±0.47		
7K7U	Catechin glucoside	2.336±0.81	1.330±0.24	5.141±0.07	257.82±38.06	381.25±2.15	371.54±6.07
	Drevogenin D	4.868±1.41	0.635±0.12	3.769±0.02	438.25±117.3	314.38±1.80	161.52±3.71
	7K7U-alone	3.215±1.39			10.14±1.60		

Note: the Angstrom unit (Å) was used to measure the mean ± SEM of the values. rGyr, radius of gyration; RMSD, root mean square deviation; RMSF, root mean square fluctuation; PSA, pressure swing adsorption; SASA, solvent-accessibility surface area; MolSA, molecular surface area.

**FIGURE 9:**
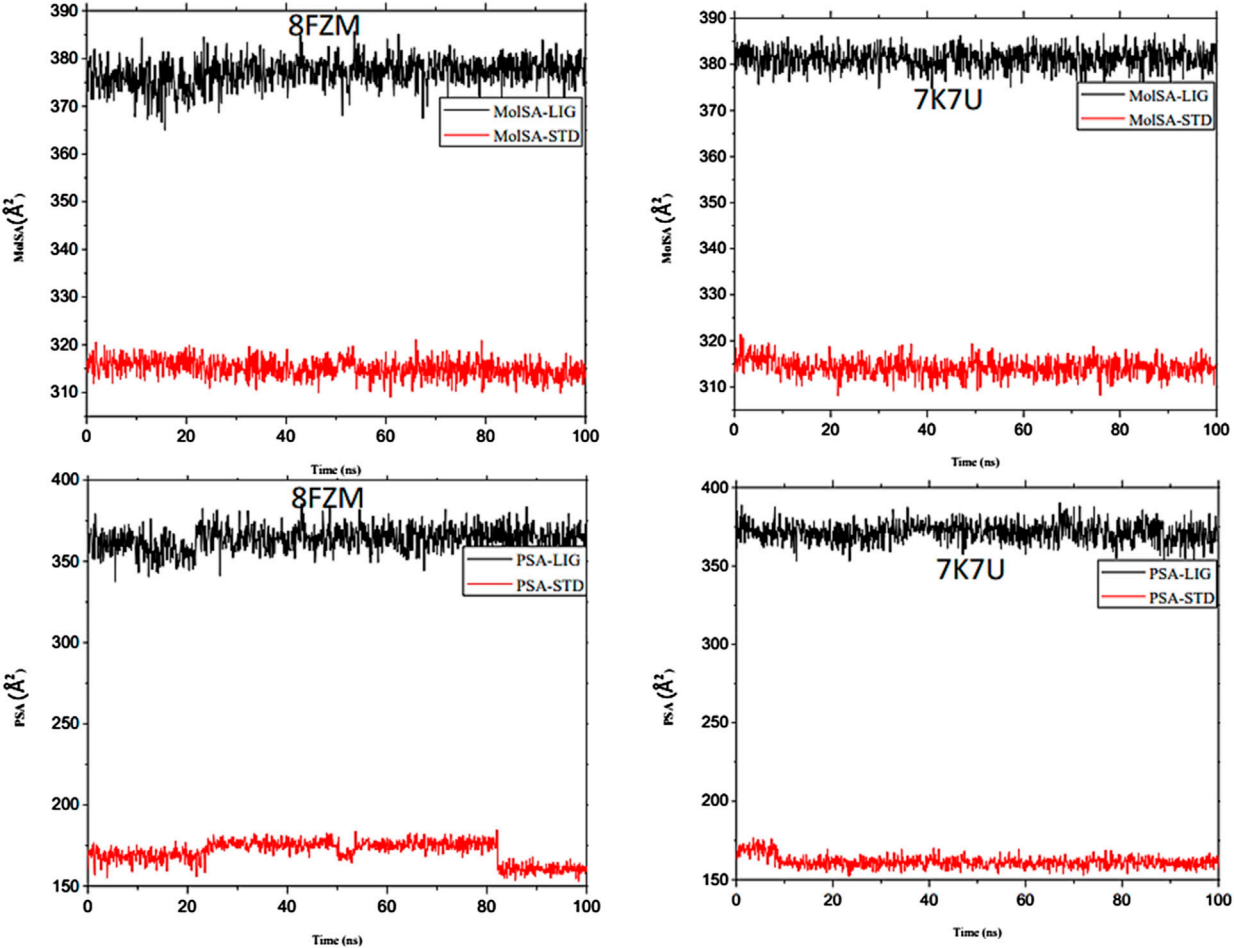
Molecular dynamics (MD) analysis of both the human importin alpha 3 activator complex and βB2-crystallin activator. Molecular surface area (MolSA) graphical illustration and PSA diagram. The structural dynamics (MD) data were analyzed using Schrödinger version 2021_1 software.

**FIGURE 10:**
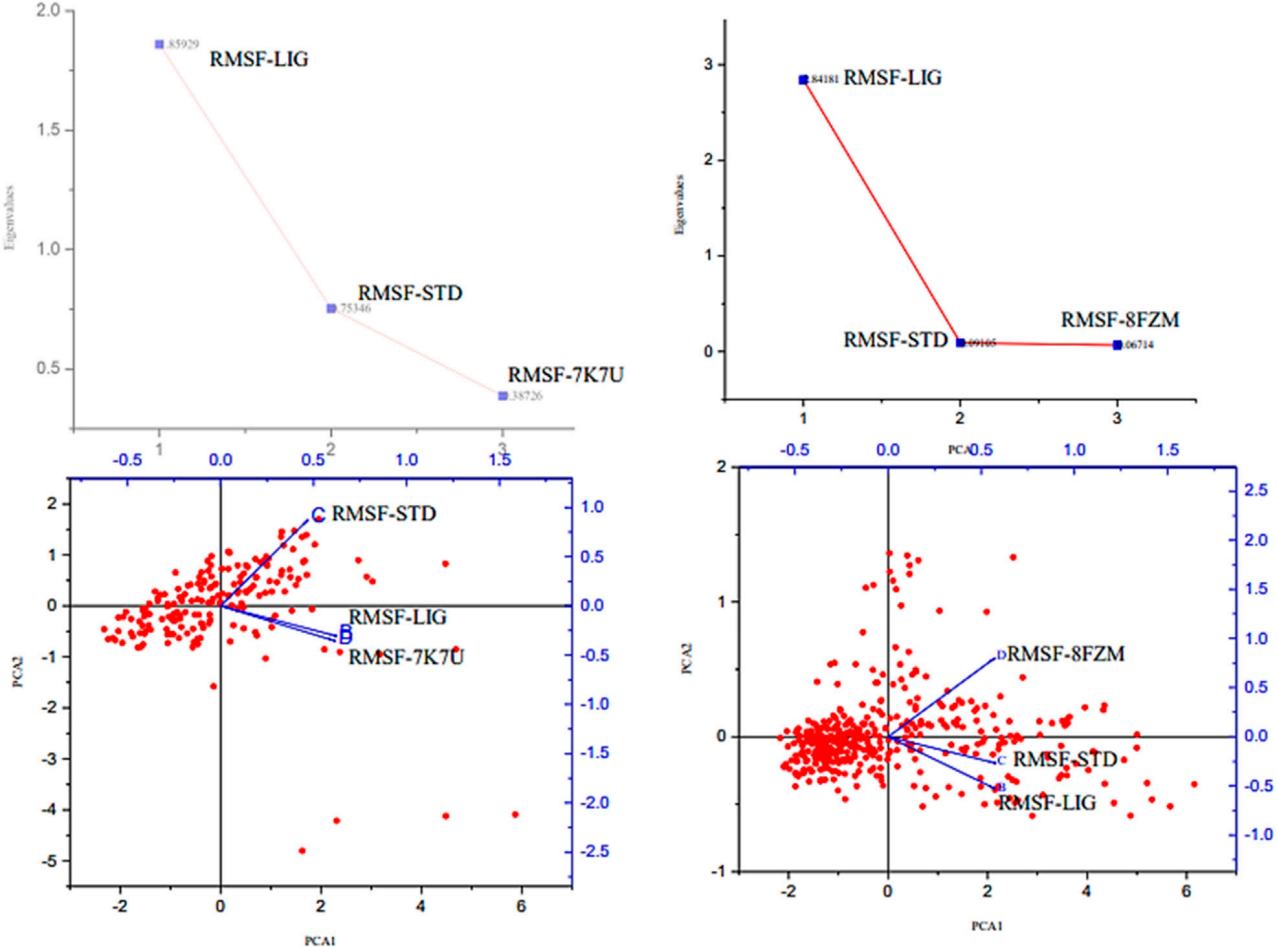
Principal component analysis of the native proteins (the human importin alpha 3 activator complex and βB2-crystallin activator) and the compound of interest based on the root mean square fluctuations.

**FIGURE 11:**
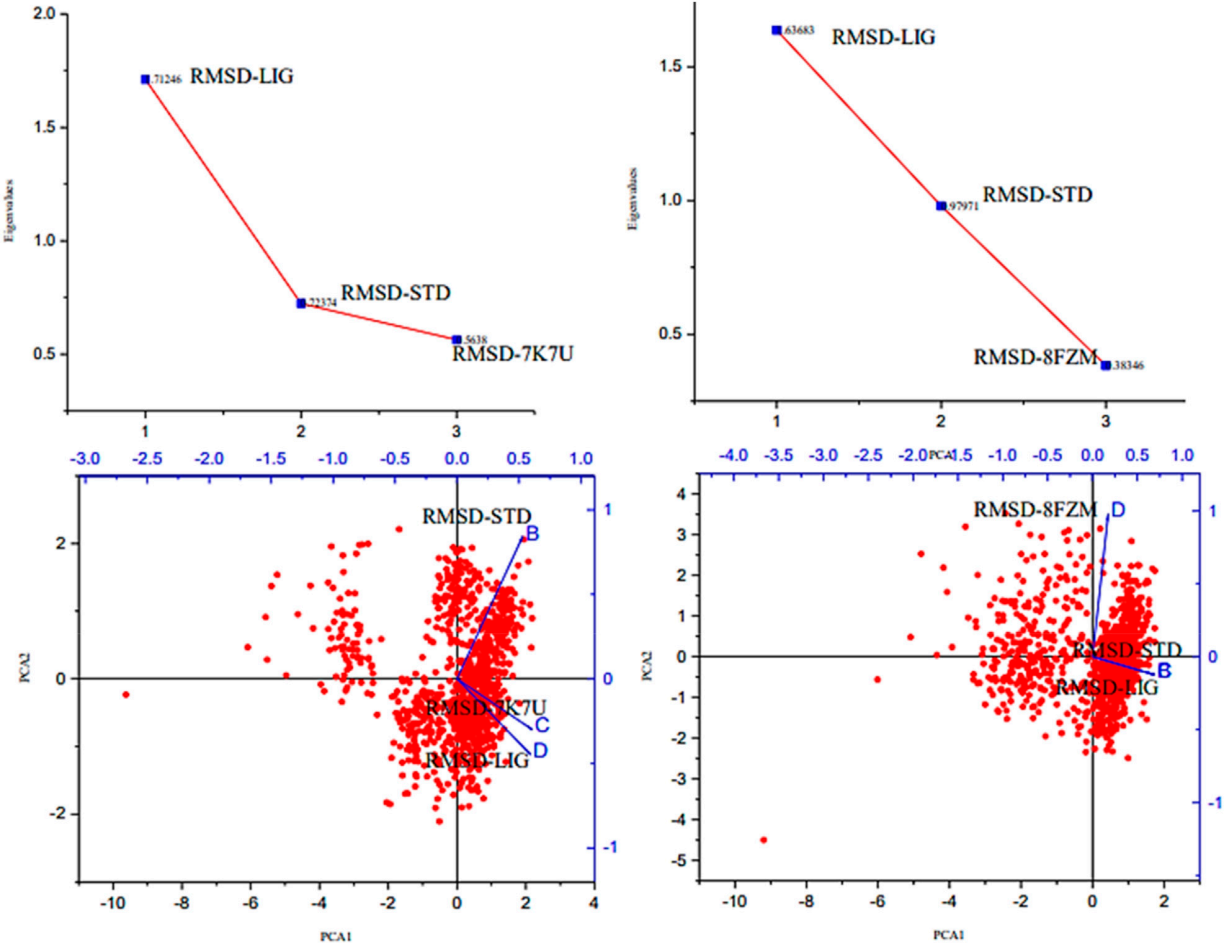
Principal component analysis of the native proteins (the human importin alpha 3 activator complex and βB2-crystallin activator) and the compound of interest based on the root mean square deviations.

The majority of MolSA alterations involve the reorganization of amino acid residues in their accessible or buried areas, which modifies the modeling of protein-ligand structures. Our investigation demonstrated the consistency of amino acid residue reorganization at both buried and accessible sites throughout the simulated period. Table 3 shows the deviations and aggressive actions, which were 377.11 ± 2.92, 381.25 ± 2.15, 315.57 ± 1.92, and 314.38 ± 1.80. A PSA study shows commensurable outcomes for almost all the molecules. Table 3 and Figure 9 provide deviation plots and mean ± SEMs for the receptor binding complex. The pattern decreases from the start to the end of the simulation. Because of the well-built synergy between human importin alpha 3 and the βB2-crystallin activator, along with atoms linked to amino acid residues in the receptor’s pose, conformational difference may increase, influencing the pose and potentially inhibiting receptors. In addition, one of the mechanics studied to better determine the major component is the stable structure of the residues in relation to a combination of linearly and uncorrelated parameters, which investigates the covariance matrix of the coordinate fluctuations in the protein simulation. Consequently, as Table 4 demonstrates, there is a considerable difference between the ligands and receptors in the scatter plot shown in Figures 10, 11. Furthermore, the eigenvalues are revealed through the diagonalization of the covariance model and the application of the OriginPro interface, as shown in both Figures 10, 11, thereby providing important information on the coordinated movements among the receptors. Finally, our study provides insights into the potential use of catechin glucoside in the treatment of cataracts. Thus, further clinical investigation is necessary to unravel the mechanistic involved and corroborate its therapeutic importance in the treatment of cataracts and associated problems.

**TABLE 4:** Principal component analysis of the native proteins and molecules of interest.

Receptor	Name	EV-cumulative	% Variance	% Cumulative	Coefficient PC1	Coefficient PC2
8FZM	Catechin glucoside	2.841	94.0	94.73	0.5777	-0.5308
	Drevogenin D	0.091	3.04	97.76	0.5793	-0.2684
	8FZM-alone	0.067	2.24	100	0.5750	+0.8038
7K7U	Catechin glucoside	1.859	61.98	61.98	0.6265	-0.3089
	Drevogenin D	0.753	25.12	87	0.4725	+0.8806
	7K7U-alone	0.387	12.91	100	0.6198	-0.3591

Keywords: EV, eigenvalue; PC, principal component; %, percentage.

## 5 Conclusion

The global focus on cataracts and their associated challenges necessitates a multi-faceted approach to mitigate their impact. Utilizing bioactive compounds derived from medicinal plants has emerged as a promising solution. In our study, we identified a new phytonutrient from *Phaseolus vulgaris* with significant inhibitory potential against cataract-related targets. Computational analysis confirmed its binding affinity without violating key drug development guidelines. This discovery holds promise for addressing inhibitory factors in disease management. However, challenges remain, particularly in optimizing docking computations and integrating biological network-based approaches. Further experimental research is needed to explore the compound’s therapeutic potential and develop effective strategies for cataract treatment.

## 6 Future directions

The findings from this study provide a computational basis for identifying bioactive compounds from *Phaseolus vulgaris* with potential anticataract properties, specifically targeting the S175G/H181Q mutation in the βB2-crystallin gene. Future research should prioritize experimental validation of these results through *in vitro* and *in vivo* studies to confirm the binding interactions and stability of the identified compounds. Biochemical assays can be employed to assess their inhibitory effects on protein aggregation and lens opacity, offering direct evidence of their therapeutic potential. Additionally, exploring the downstream molecular pathways affected by the S175G/H181Q mutation and understanding how these compounds modulate oxidative stress and protein misfolding could provide deeper mechanistic insights.

The evaluation of the biological activity of these compounds in targeted assays is crucial to strengthen the computational predictions and identify the most promising candidates for further development. This will also provide the foundation for exploring their pharmacokinetics and safety profiles in preclinical models. Subsequent clinical trials would be essential to determine their therapeutic efficacy and applicability in preventing or treating cataracts.

To broaden the scope of this research, future investigations could focus on other mutations in the βB2-crystallin gene associated with cataract formation, thereby expanding the potential targets for these bioactive compounds. Moreover, extending the analysis to compounds from related medicinal plants known for their anticataract properties might uncover additional therapeutic options. Integrating these findings with network-based approaches and clinical research will ultimately pave the way for developing effective strategies to mitigate cataracts and improve patient outcomes.

## Data Availability

The original contributions presented in the study are included in the article/Supplementary Material; further inquiries can be directed to the corresponding author.
