# Supplementary figures and images for: In silico identification of the anticataract target of βB2-crystallin from Phaseolus vulgaris: a new insight into cataract treatment
Source: Front Chem. 2025 Jan 17;12:1421534. doi: 10.3389/fchem.2024.1421534 (PMC11782562; doi:10.3389/fchem.2024.1421534)

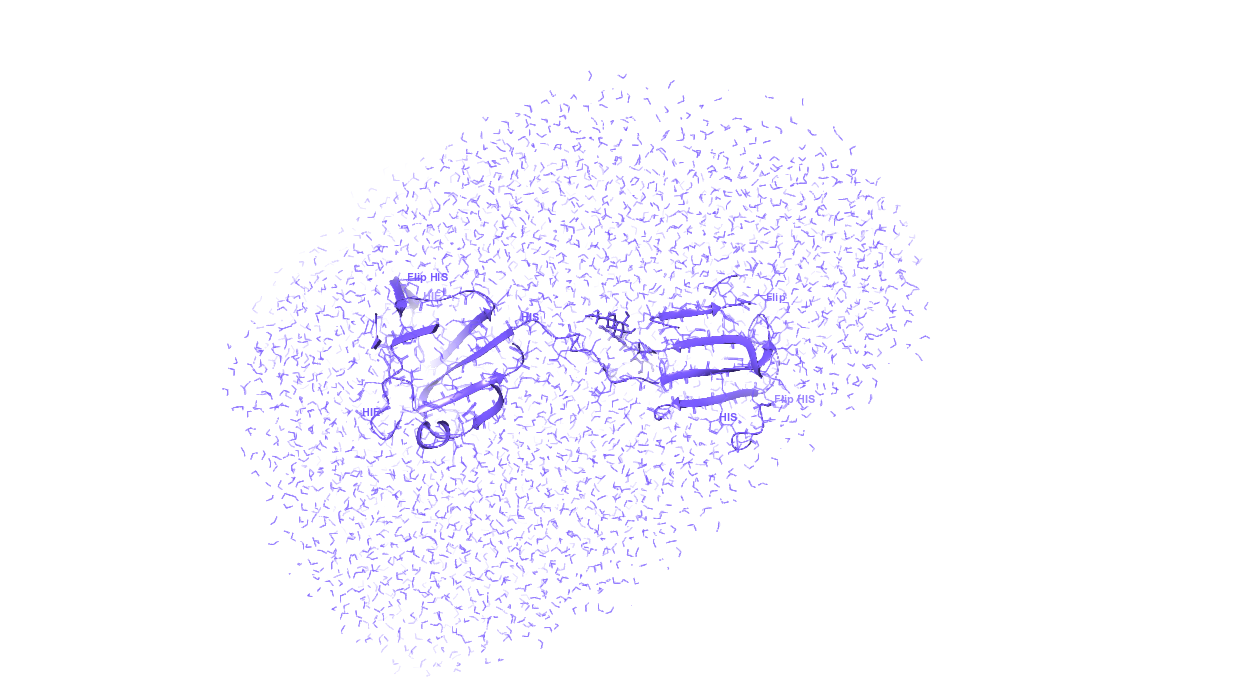

Supplement: Supplementary file 1 [file Image6.tif]

## Slide 1
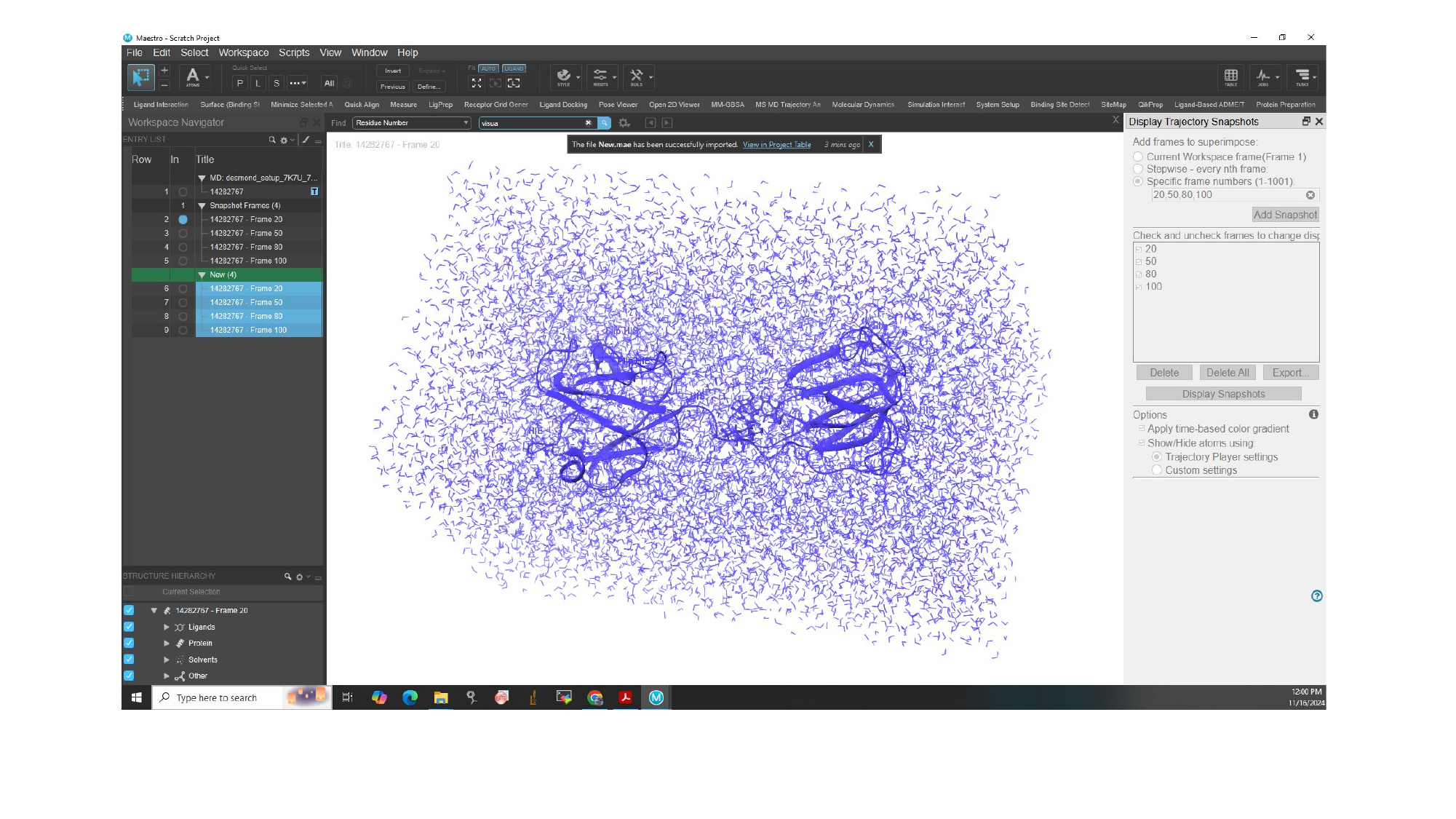

Supplement: Supplementary file 2 [file Presentation1.pptx]

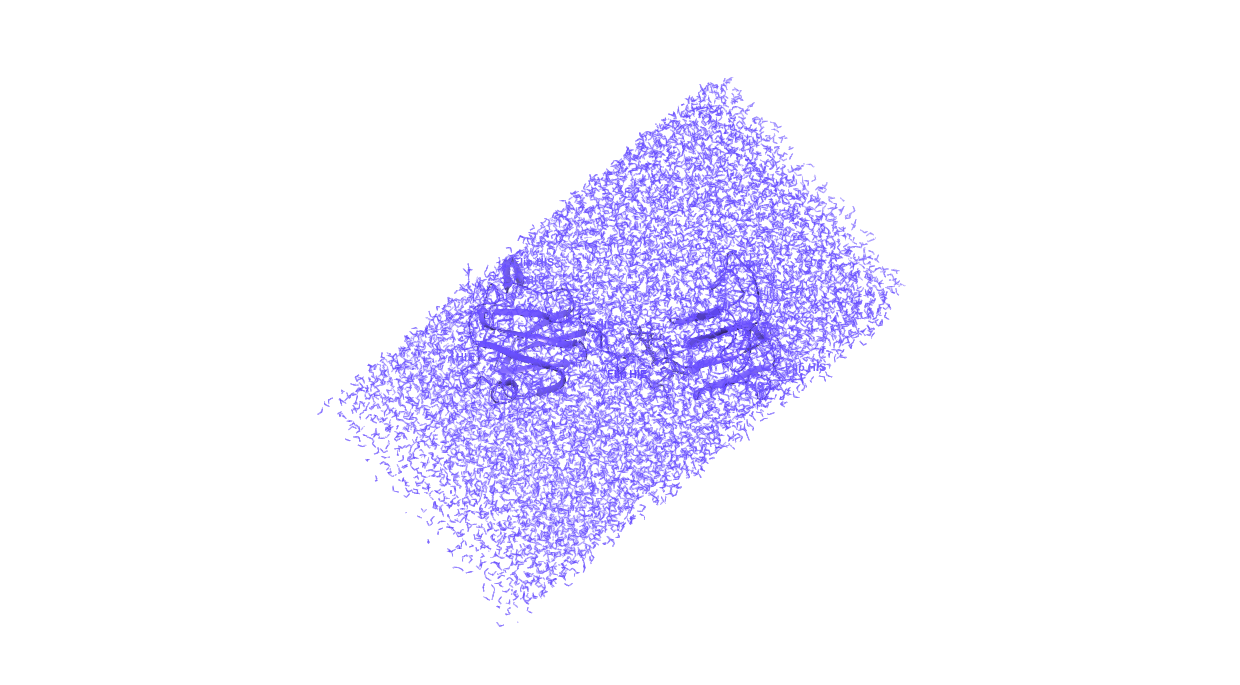

Supplement: Supplementary file 3 [file Image3.tif]

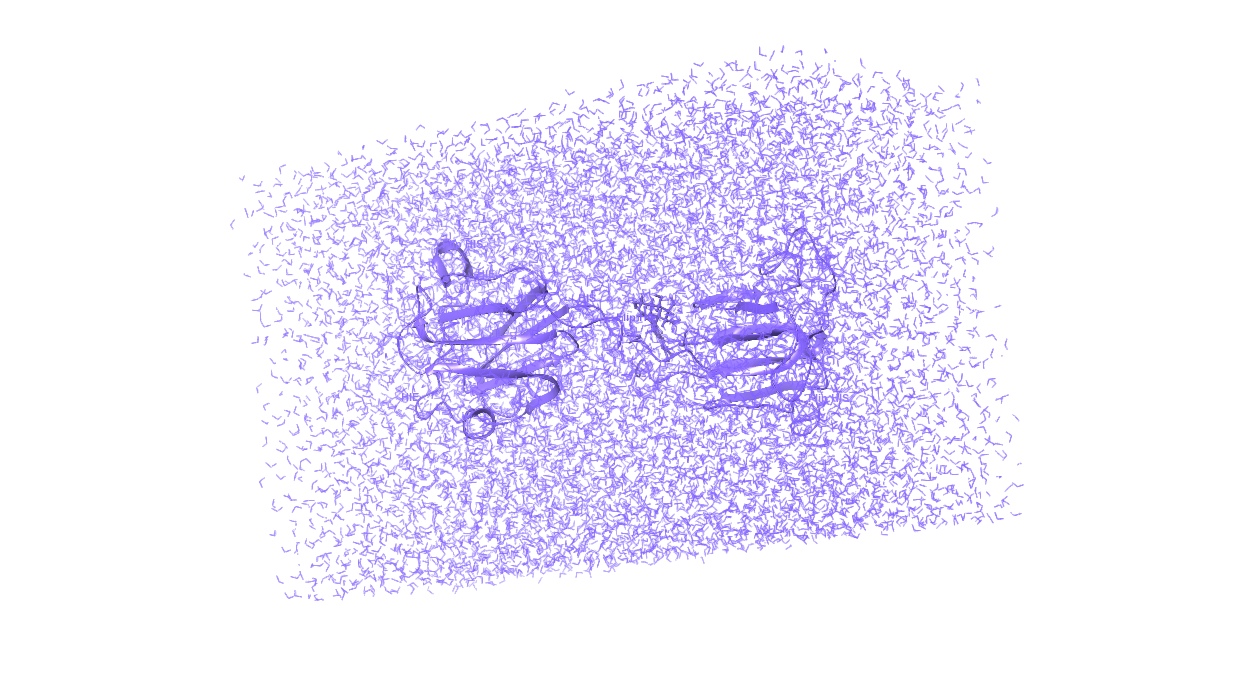

Supplement: Supplementary file 4 [file Image4.tif]

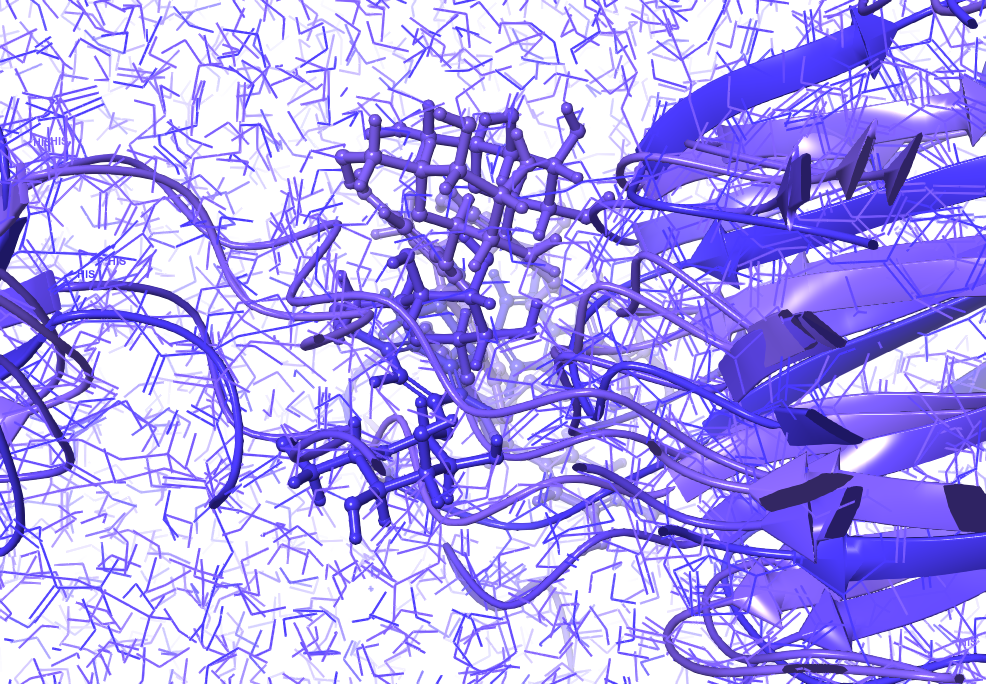

Supplement: Supplementary file 5 [file Image1.tif]

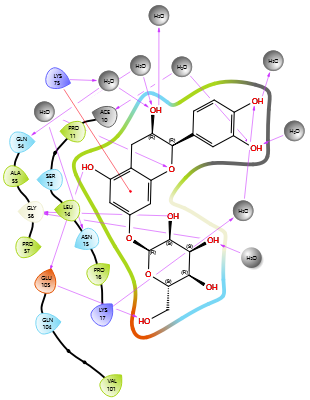

Supplement: Supplementary file 6 [file Image2.png]

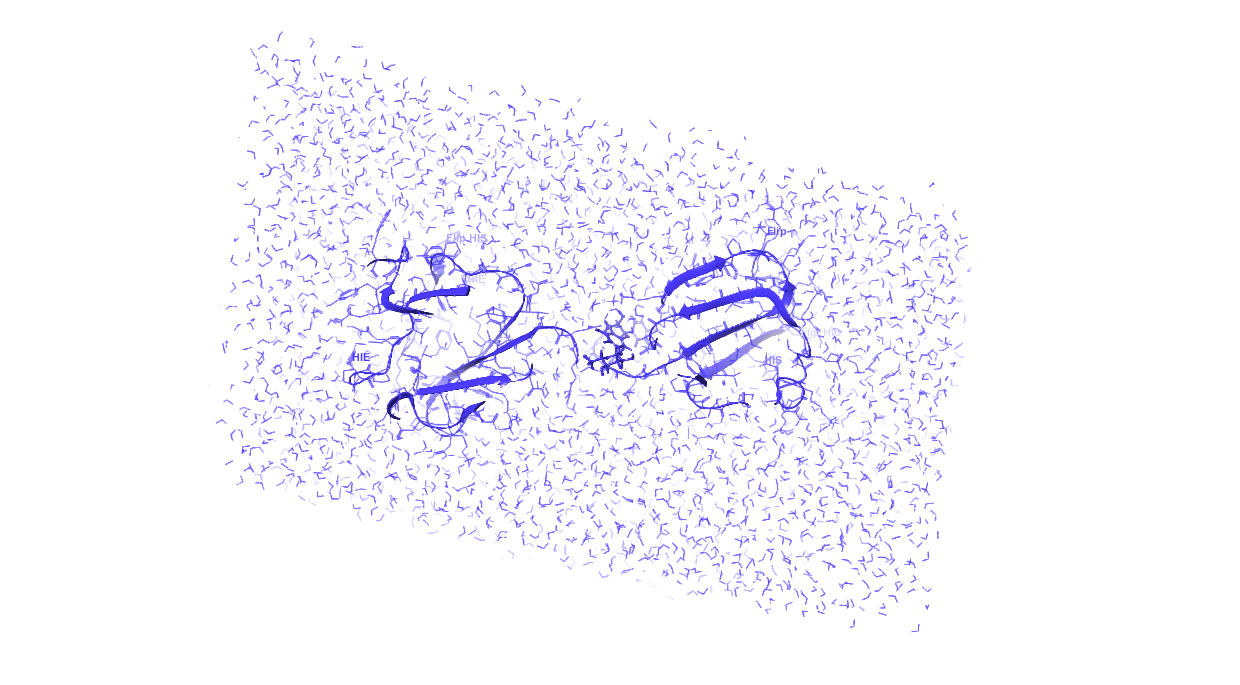

Supplement: Supplementary file 7 [file Image5.tif]
