# Supplementary material for: In silico identification of the anticataract target of βB2-crystallin from Phaseolus vulgaris: a new insight into cataract treatment
Source: Front Chem. 2025 Jan 17;12:1421534. doi: 10.3389/fchem.2024.1421534 (PMC11782562; doi:10.3389/fchem.2024.1421534)
Supplement: Supplementary file 8 [file DataSheet1.docx]

**7K7U_STD** Simulation interactions diagram report

Protein-ligand interactions are categorized into four types: Hydrogen Bonds, Hydrophobic, Ionic and Water Bridges. Each interaction type contains more specific subtypes, which can be explored through the 'Simulation Interactions Diagram' panel.


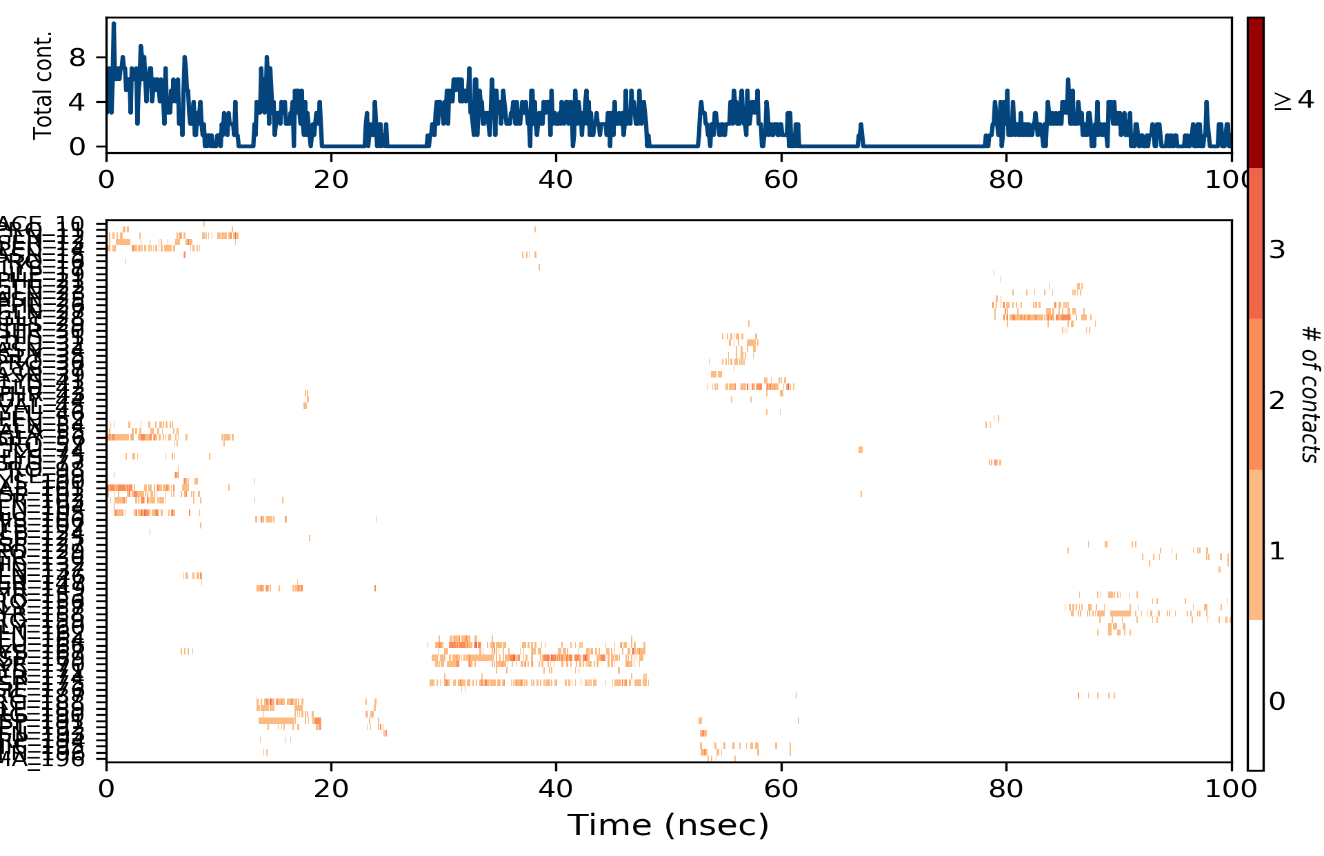


A timeline representation of the interactions and contacts (**H-bonds**, **Hydrophobic**, **Ionic**, **Water bridges**) summarized in the previous page. The top panel shows the total number of specific contacts the protein makes with the ligand over the course of the trajectory. The bottom panel shows which residues interact with the ligand in each trajectory frame

**7K7U_Lig** Simulation interactions diagram report


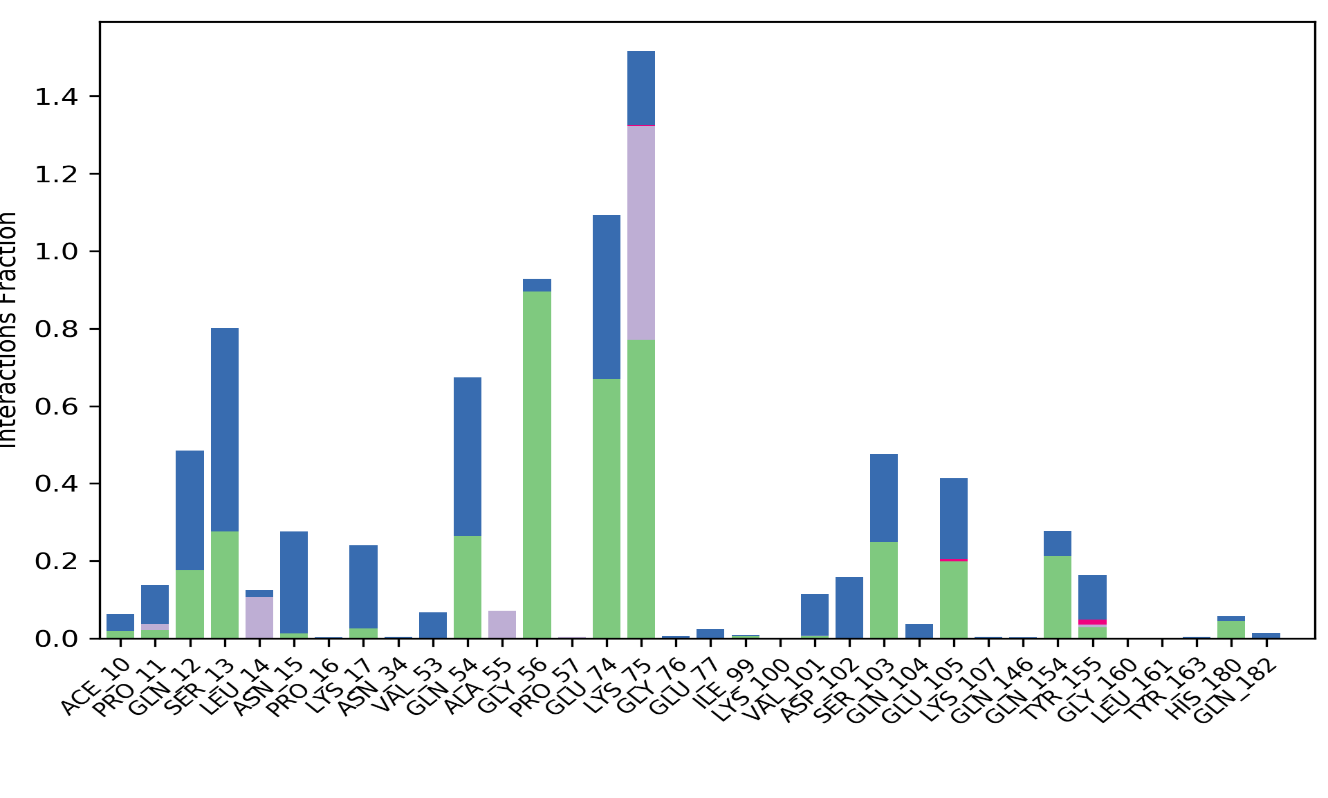

Protein-ligand interactions are categorized into four types: Hydrogen Bonds, Hydrophobic, Ionic and Water Bridges. Each interaction type contains more specific subtypes, which can be explored through the 'Simulation Interactions Diagram' panel


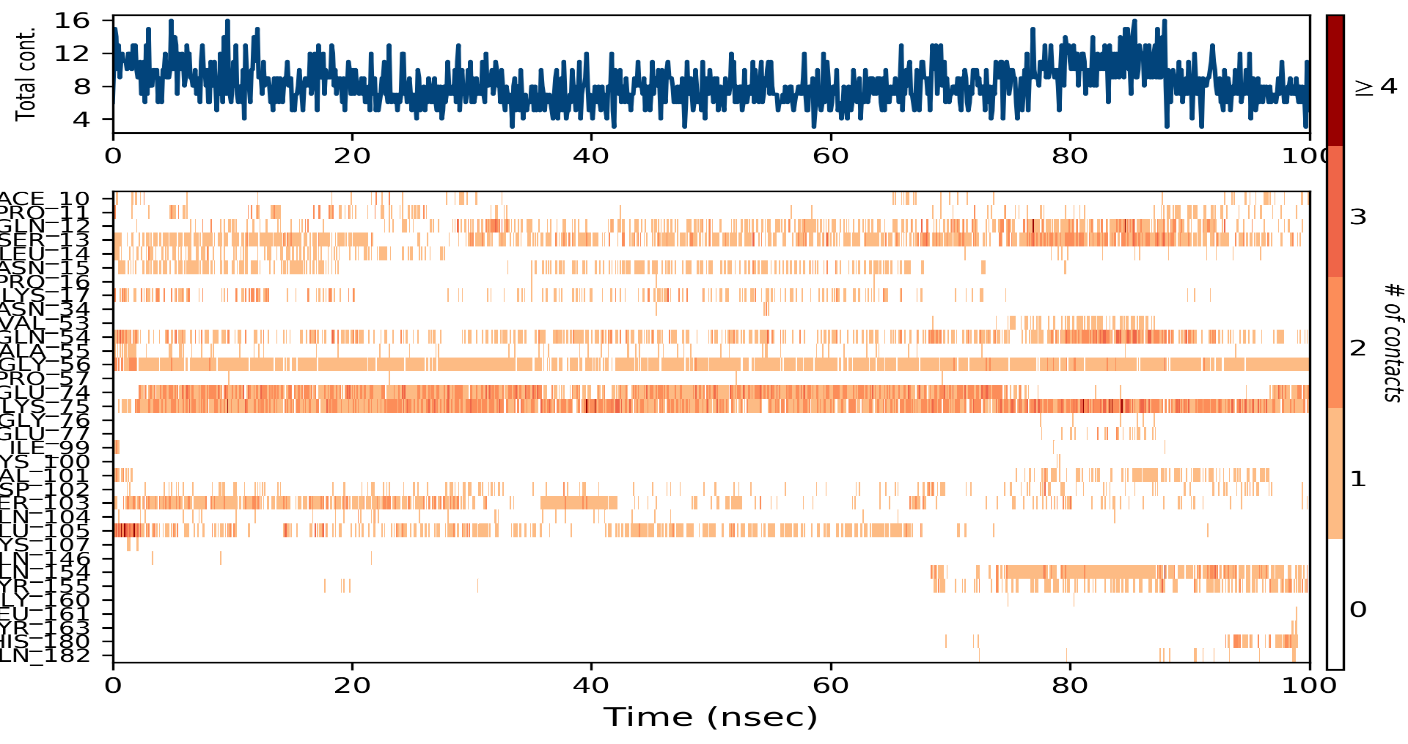


A timeline representation of the interactions and contacts (**H-bonds**, **Hydrophobic**, **Ionic**, **Water bridges**) summarized in the previous page. The top panel shows the total number of specific contacts the protein makes with the ligand over the course of the trajectory. The bottom panel shows which residues interact with the ligand in each trajectory frame

**8fzm_STD** Simulation interactions diagram report


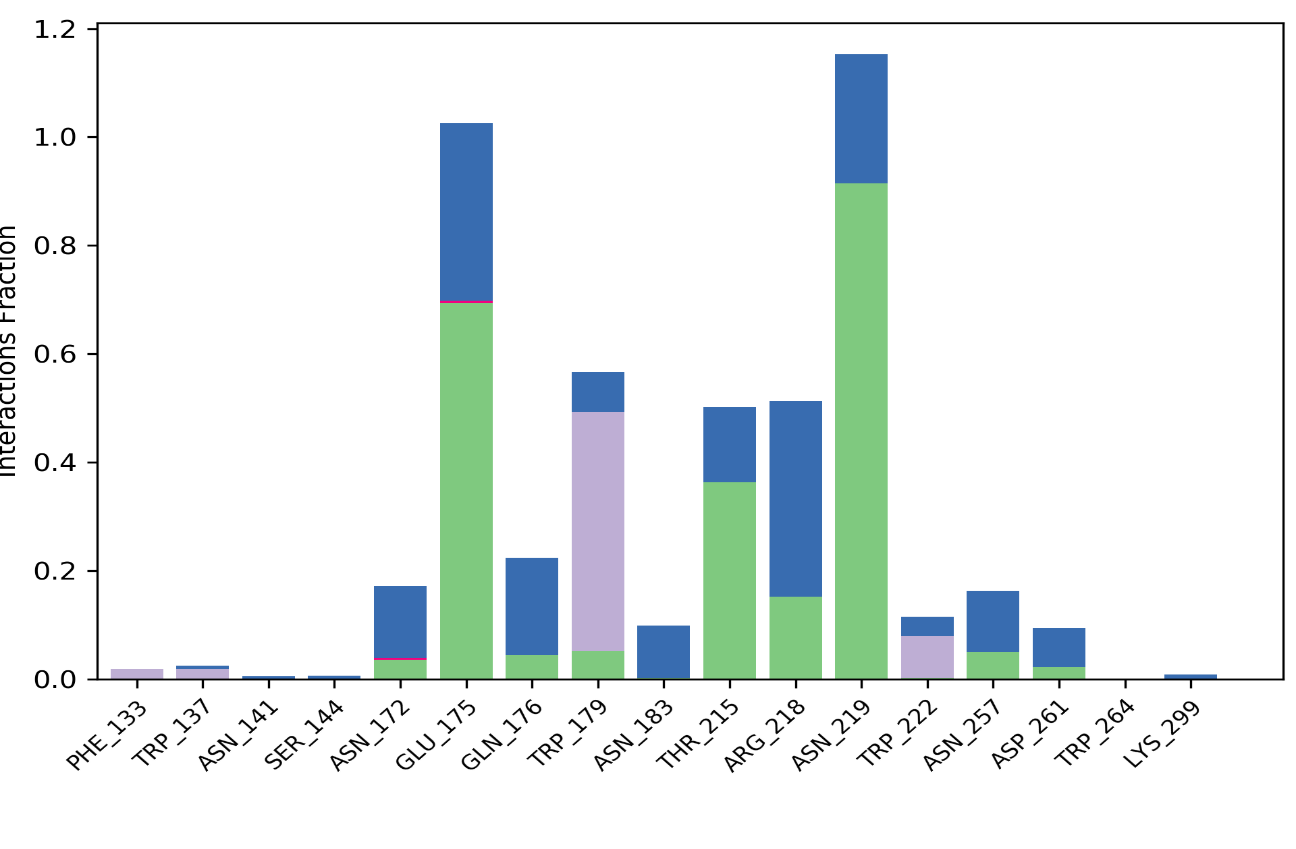

Protein-ligand interactions are categorized into four types: Hydrogen Bonds, Hydrophobic, Ionic and Water Bridges. Each interaction type contains more specific subtypes, which can be explored through the 'Simulation Interactions Diagram' panel


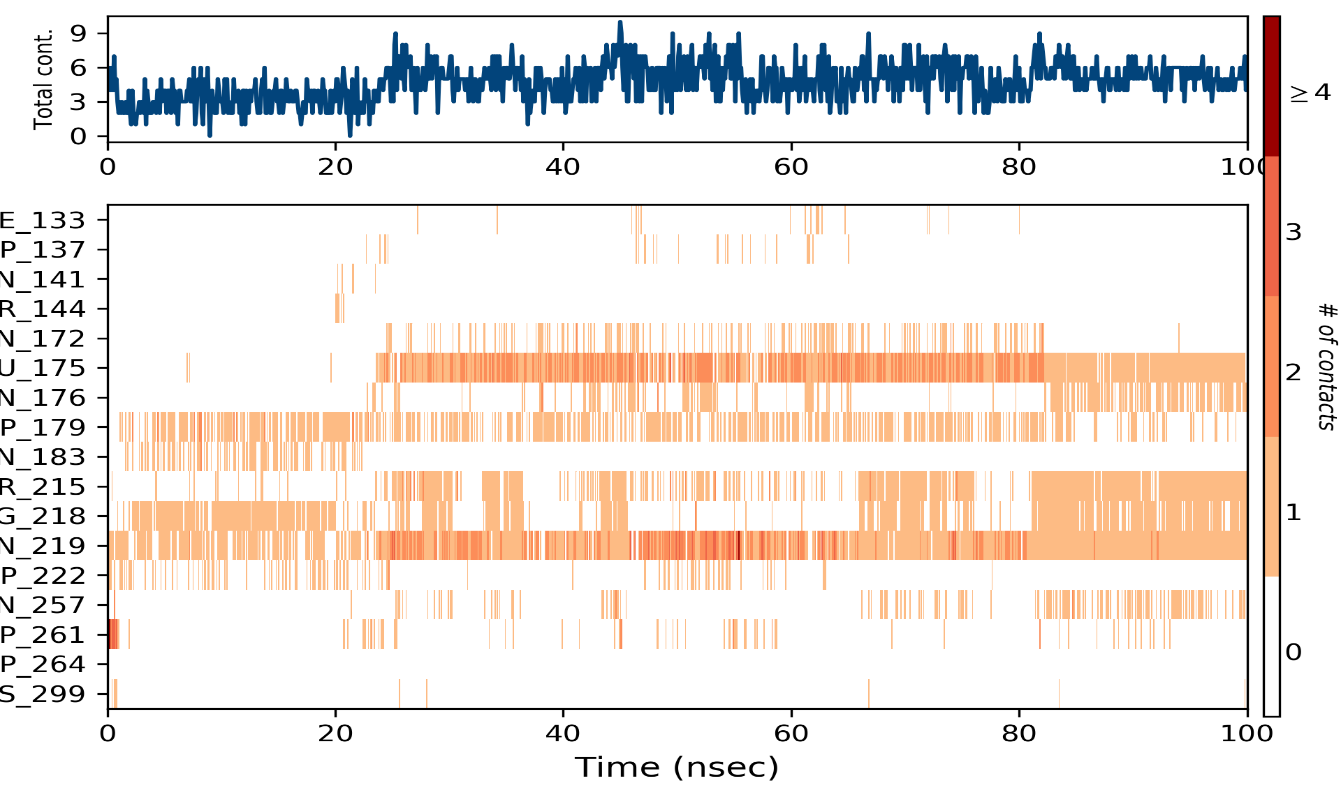


A timeline representation of the interactions and contacts (**H-bonds**, **Hydrophobic**, **Ionic**, **Water bridges**) summarized in the previous page. The top panel shows the total number of specific contacts the protein makes with the ligand over the course of the trajectory. The bottom panel shows which residues interact with the ligand in each trajectory frame
